# A Serine/Threonine Kinase 16-Based Phospho-Proteomics Screen Identifies WD Repeat Protein-1 As A Regulator Of Constitutive Secretion

**DOI:** 10.1038/s41598-018-31426-1

**Published:** 2018-08-29

**Authors:** Alfonso López-Coral, Anneliese C. Striz, Pamela L. Tuma

**Affiliations:** Department of Biology, The Catholic University of America, Washington DC, 20064 USA

## Abstract

The plasma membrane of polarized hepatocytes is functionally divided into two domains: the apical and basolateral. Our focus is to define the molecular basis of polarized protein sorting of newly-synthesized membrane and secretory proteins in WIF-B cells, an excellent model system for polarized hepatocytes. We determined that MAL2 (myelin and lymphocyte protein 2) and its binding partner, serine/threonine kinase 16 (STK16) regulate basolateral constitutive secretion. Because STK16 is a constitutively active kinase, we reasoned that constitutively phosphorylated substrates must participate in constitutive secretion. To identify either STK16 substrates or other proteins that regulate constitutive secretion, we took a proteomics approach. Post-nuclear supernatants from cells expressing wild type or a kinase-dead (E202A) STK16 were separated on 2D gels and immunoblotted with antibodies against phospho-serine/threonine residues. Sixteen spots were identified from E202A-expressing cells that reproducibly displayed decreased immunoreactivity. From these spots, 28 proteins were identified as possible STK16 substrates. Out of these 28 possible substrates, 25% of them encode predicted STK16 phosphorylation consensus sites, with WD repeat containing protein-1 (WDR1) encoding two such sites. Based on this finding and on the finding that actin remodeling is required for hepatic secretion, we further confirmed that WDR1 is a phosphoprotein that regulates secretion.

## Introduction

The hepatocyte surface is functionally divided into two domains: the basolateral and apical. The basolateral surface faces the blood whereas the apical surface faces the bile, the complex molecular soap needed for dietary fat absorption and waste removal. This functional “polarity” is mirrored by the asymmetrical distribution of proteins and lipids required for proper liver function. Our research addresses the fundamental question of how such hepatic polarity is established and maintained. Our focus has been to define the molecular mechanisms and identify the molecules that regulate polarized protein sorting of newly-synthesized membrane and secretory proteins in polarized hepatic WIF-B cells.

WIF-B cells are an excellent hepatic model system for the study of liver in health and in disease. They enter a terminal differentiation program, and after 7–10 days in culture, ~90% of cells are fully differentiated and exhibit polarized surface domains that are functionally and compositionally analogous to the apical and basolateral surfaces^1^. Importantly for these studies, polarized protein sorting/trafficking pathways are conserved as they are in hepatocytes *in situ*. We have characterized WIF-B endocytic and biosynthetic pathways and mapped out the itineraries of recycling receptors, apical and basolateral residents^2–6^ and have developed many useful assays for monitoring protein trafficking^7^. With such assays in hand, the WIF-B cells have been used as surrogates for normal hepatocytes by us and others in studies of protein trafficking and activity, bile acid and organic ion secretion, phospholipid translocation and HDL biosynthesis^8–19^. Also importantly, liver specific activities are maintained in culture as they are *in situ* such that WIF-B cells have also been used to examine many types of liver injury including drug-, heavy metal-, hypoxia- and alcohol-induced injury, steatosis, sitosterolemia, hemochromatosis, atherosclerosis, familial intrahepatic cholestasis, hypercholesterolemia and Wilson’s Disease^20–30^.

Over a decade ago, we initiated studies to examine the role of MAL2 (myelin and lymphocyte protein 2) in regulating hepatic polarized protein sorting. We first confirmed that MAL2 functioned in transcytosis in WIF-B cells^31^. Like in HepG2 cells^32^, we determined that transport from the early endosome to the sub-apical compartment (SAC) of three classes of newly-synthesized apical residents was impaired: polymeric IgA-receptor (pIgA-R), single spanning and GPI-anchored residents^31^. In the course of those studies, we further determined that MAL2 selectively regulates delivery of newly-synthesized pIgA-R (but *not* other single spanning apical proteins) from the TGN to the basolateral membrane. And most recently, we determined that MAL2 also regulates constitutive secretion of soluble cargo along with its binding partner, serine/threonine kinase 16 (STK16)^33^. Because little is known about the molecular machinery that regulates constitutive secretion, we have continued our analysis of STK16.

STK16 belongs to the unique, small family of numb-associated kinases. These divergent kinases share less than 25% identity with their nearest neighbor and are not well characterized. STK16 is a particularly interesting and enigmatic member of this family. Out of its 305 amino acids, only 29 are outside the catalytic site, the first 19 and the last 10 amino acids^34^. STK16 encodes no regulatory domains and is constitutively active^34^. Based on our previous results of the overexpression of a dominant negative, kinase-dead version of the enzyme, we determined that kinase activity is required for STK16 function in secretion^33^. This further implies that constitutively phosphorylated substrates must also participate in constitutive secretion.

To identify additional molecular players that either participate in secretion directly or are part of a kinase/phosphatase network that regulates secretion, we took a proteomics approach. Post-nuclear supernatants from polarized hepatic WIF-B cells expressing wild type or the kinase-dead STK16 (E202A) were separated on 2D gels and immunoblotted with antibodies against phospho-serine/threonine residues. In all, 98 proteins were identified. After removing duplicate hits, 68 proteins were further considered. Based on the finding that actin remodeling is required for hepatic secretion^35^, we further examined WD repeat-containing protein 1(WDR1) 1 as a potential component of the molecular machinery that drives constitutive secretion or as a part of a STK16-mediated phosphoprotein network that regulates secretion.

## Results

### A phospho-proteomic screen identifies possible STK16 substrates and/or regulators of constitutive secretion

**Figure 1:**
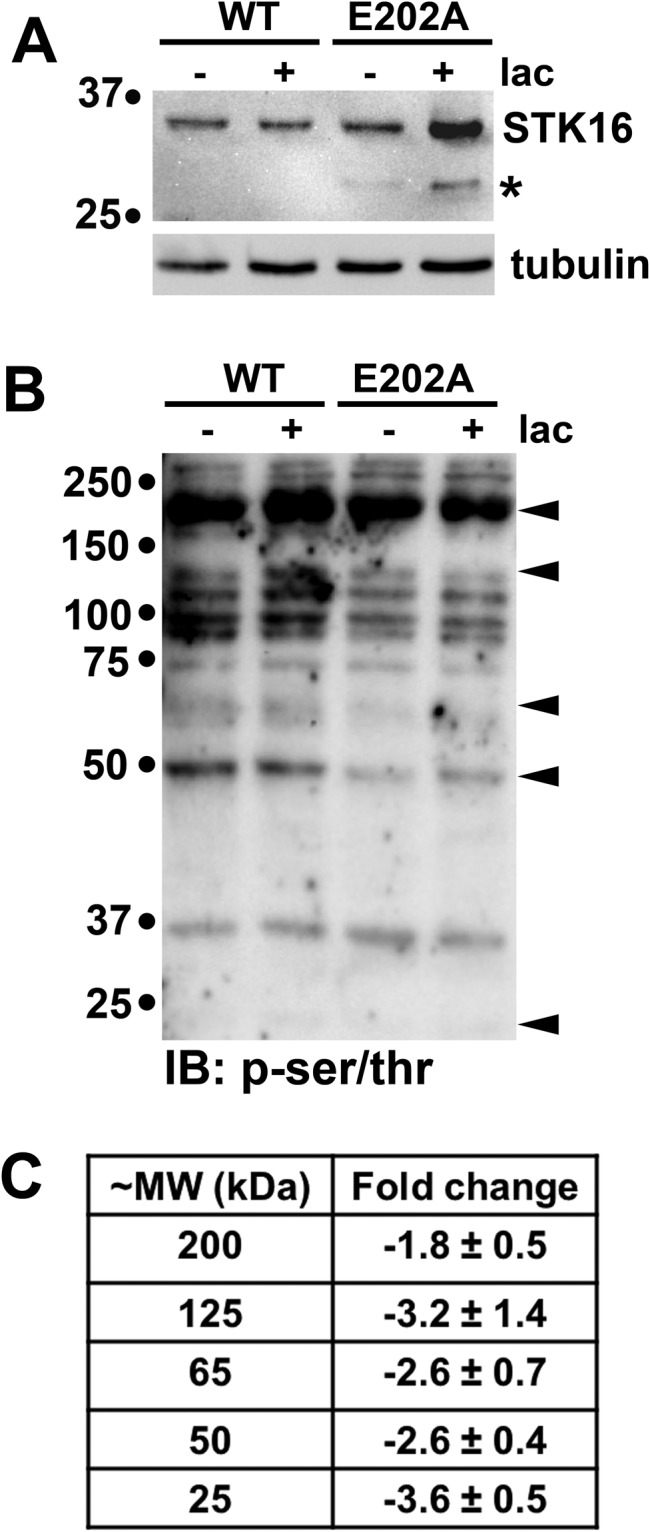
E202A expression leads to decreased serine/threonine phosphorylation in total cell lysates. (**A**) Total cell lysates were prepared from WIF-B cells expressing wild type (WT) or the kinase dead STK16 (E202A) after treatment in the absence or presence of 5 μM lactacystin (lac) for 4 h at 37 °C. Lysates (20 μg total protein per lane) were immunoblotted for STK16 (with anti-V5 antibodies) (top panel) or α-tubulin (as a loading control). The asterisk marks a known degradative species of the mutant kinase. Molecular weight standards are indicated on the left (in kDa). (**B**) The lysates as in A were immunoblotted (IB) with antibodies against phosphorylated-serine/threonine residues (p-ser/thr). Molecular weight standards are indicated on the left (in kDa) and arrows on the right indicate proteins with decreased immunoreactivity in E202A expressing cells. (**C**) The fold-decrease in immunoreactivity of the proteins marked with arrows in B (from samples without lactacystin) was determined using densitometry. Values represent the average from three independent experiments ± SEM.

To identify STK16 substrates and implicate additional molecular players that function in secretion, we took a proteomics approach. Because our strategy relied on detecting decreased protein phosphorylation levels in E202A expressing cells, we specifically chose a 2D gel immunoblotting/LC-MS/MS approach. Although admittedly less sensitive and precise than mass spectrometry alone, it allowed us to easily visualize decreased detection levels in order to select proteins for further analysis. As a proof of principle experiment for our approach, cells expressing wild type or the kinase-dead (E202A) STK16 were washed and lysed in buffers containing sodium vanadate and phosphatase inhibitors and 20 μg of total protein from each sample were immunblotted with antibodies specific for phospho-serine and -threonine residues. Cells were also pre-treated for 4 h in the absence or presence of the proteasome inhibitor, lactacystin, a treatment we have shown enhances E202A expression^33^. As shown in Fig. 1A, both forms of the ~35 kDa kinase were robustly expressed in WIF-B cells with enhanced detection of E202A in cells treated with lactacystin. Tubulin levels served as the loading control. In Fig. 1B, decreased immunoreactivity of a number of discrete bands (marked with arrows) was observed in E202A expressing cells on immunoblots performed in parallel. Further decreases were seen in E202A expressing cells treated with lactacystin. To quantitate the decreased immunoreactivity of selected bands in E202A expressing cells, we prepared whole cell lysates prepared in the absence of lactacystin to avoid confounding results due to proteosome inhibition. As shown in Fig. 1C, reproducible decreases from ~2.0 to 3.5-fold were observed for all selected species, varying from high to low molecular weight, confirming the feasibility of our approach.

**Figure 2:**
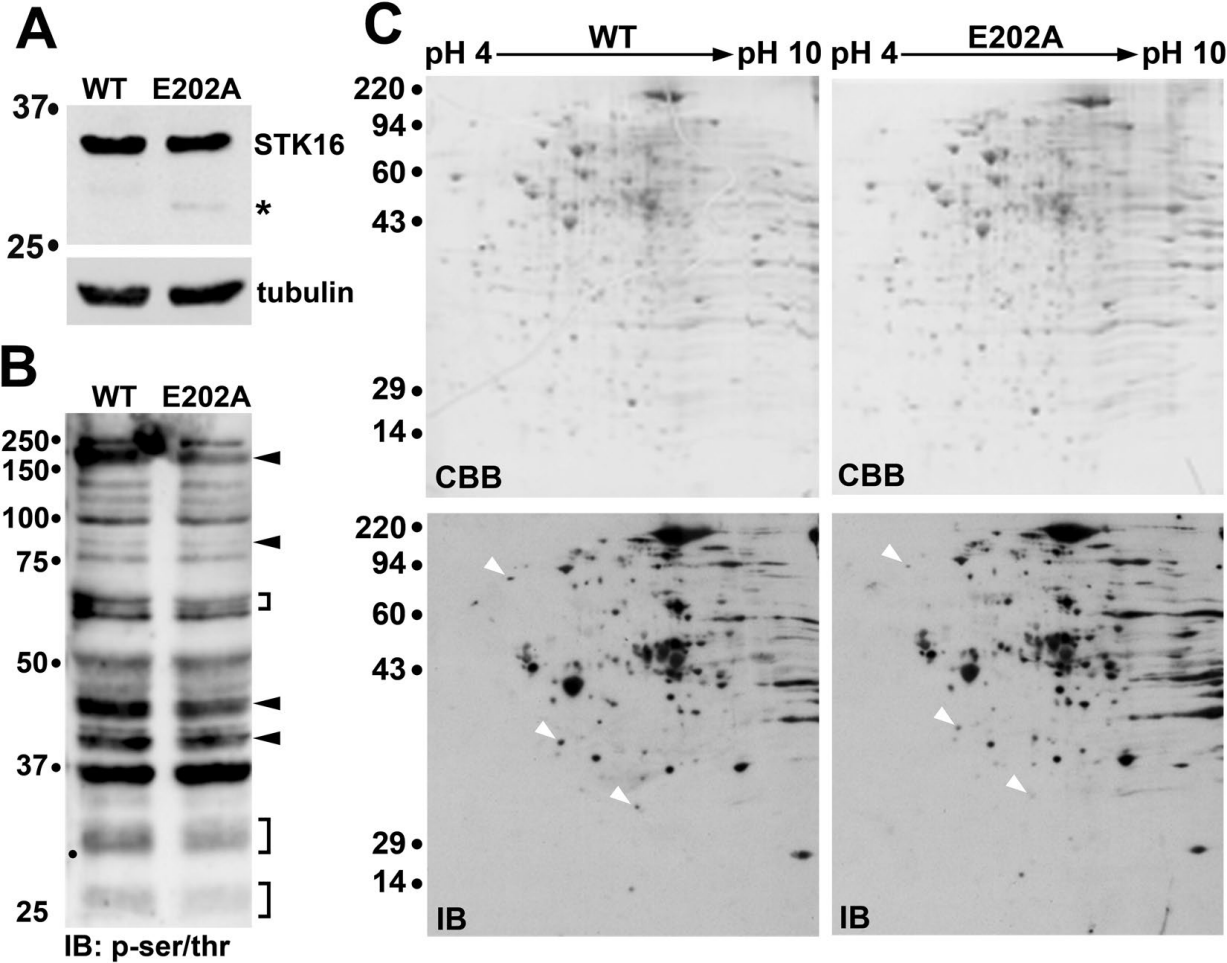
E202A expression leads to decreased serine/threonine phosphorylation in post-nuclear supernatants. (**A**) Post nuclear lysates (20 μg total protein per lane) prepared from WIF-B cells expressing wild type (WT) or the kinase dead STK16 (E202A) were immunoblotted for STK16 (with anti-V5 antibodies) (top panel) or α-tubulin (as a loading control). The asterisk marks a known degradative species of the mutant kinase. Molecular weight standards are indicated on the left (in kDa). (**B**) The same lysates as in A were immunoblotted (IB) with antibodies against phosphorylated-serine/threonine residues (p-ser/thr). Molecular weight standards are indicated on the left (in kDa), arrows and brackets on the right indicate proteins with decreased immunoreactivity in E202A expressing cells. (**C**) Post-nuclear lysates were prepared from WIF-B cells expressing wild type (WT) or E202A. 550 μg of total protein per gel were separated by 2D electrophoresis and immunoblotted with anti-phospho-serine/threonine antibodies. The pH gradient of the first dimension is indicated across the top and the molecular weight standards are indicated on the left in kDa. The Coomassie blue stained gels (CBB) are shown in the upper panels and the corresponding immunoblots (IB) are shown below. Samples were processed in duplicate (technical replicates). A representative pair from each condition is shown. Arrowheads mark three examples of proteins with decreased immunoreactivity in E202A expressing cells.

Because nuclear proteins are not likely STK16 substrates involved in secretion, we further analysed 20 μg of total protein from post-nuclear supernatants prepared from cells expressing wild type or E202A STK16 for differential phosphorylation. As for Fig. 1C, we chose not to include lactacystin. As shown in Fig. 2A, robust and equivalent levels of both the wild type and E202A STK16 were detected in these samples. Additionally, decreased detection of a number of discrete bands (marked with arrows) and clusters of bands (marked with brackets) was observed in E202A expressing cells (Fig. 2B).

To identify possible STK16 substrates and other members of the phospho-network in an as unbiased way as possible, we further analyzed the post-nuclear supernatants by 2D PAGE followed by LC-MS/MS. 550 μg of protein from wild type or E202A expressing cell lysates were resolved on 2D gels in duplicate and immunoblotted with the anti-phospho-serine/threonine antibodies. Coomassie Blue-stained gels revealed that the gels were equally loaded and displayed similar staining patterns (Fig. 2C, CBB; only one of the technical duplicates is shown). Although the overall number of spots did not significantly differ on the immunoblots, closer examination revealed differential patterns of immunoreactivity on blots from E202A expressing cells (Fig. 2C,IB). Arrowheads mark three examples of proteins with decreased immunoreactivity on immunoblots from E202A expressing cells.

**Figure 3:**
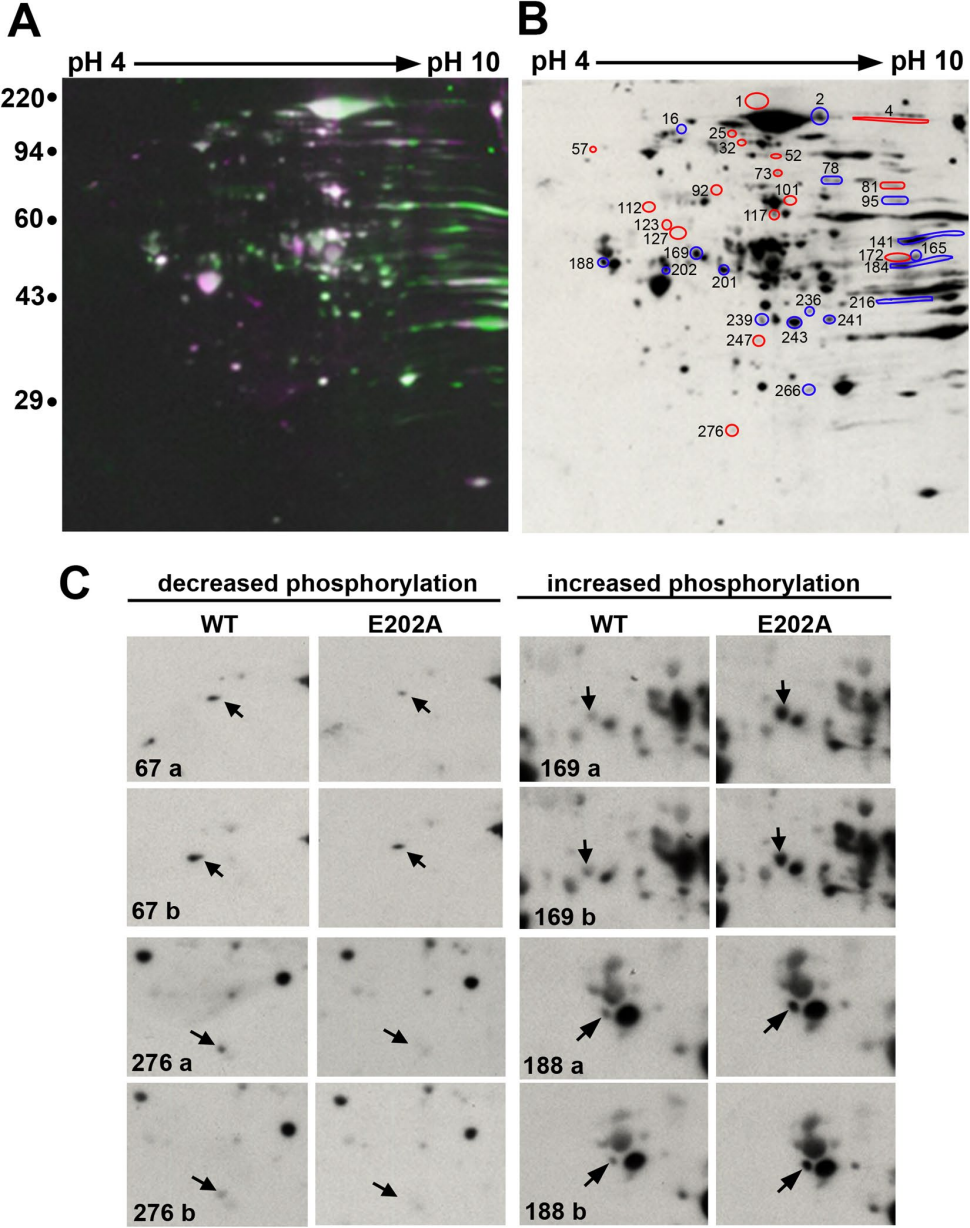
Numerous proteins display differential and reproducible phosphorylation in E202A expressing cells. (**A**) Images of the immunoblots from control and E202A expressing cells were merged and pseudo-colored. White regions indicate overlapping spots. The pH gradient of the first dimension is indicated across the top and the molecular weight standards are indicated on the left in kDa. (**B**) The overlapping spots were analyzed by densitometry (see Table 1). The spots with altered immunoreactivity were numbered. Those circled in red are those proteins with decreased immunoreactivity whereas those circled in blue, had increased levels. The pH gradient of the first dimension is indicated across the top and the molecular weight standards are indicated on the left in kDa. Experiments in A and B were performed in duplicate. A representative merged image is shown in A, and a representative immunoblot of E202A expressing cells is shown in B. (**C**) Two examples of enlarged regions from duplicate immunoblots displaying (left panels) or increased immunoreactivity (right panels) from E202A expressing cells that were selected for mass spectrometric analysis are shown. Arrows point to the specific spots and the spot numbers are indicated. Only those spots that showed similar changes in intensity for both sets were selected for further analysis.

To identify spots with altered immunoreactivity, images of blots with lysates from wild type STK16 expressing cells were merged with the blots with samples from E202A expressing cells (Fig. 3A, only one merged image is shown). From the merged images, only the discrete spots (in white) were selected for analysis while the many smeared regions were excluded. To determine the fold-change in phosphorylation, the density of individual spots on the gels and immunoblots was determined. The level of each of the immunoreactive spots was normalized to the relative protein level of its corresponding spot in the Coomassie blue-stained gel. The fold-change in phosphorylation was calculated by comparing wild type ratios to those from E202A expressing cells. In all, 282 spots were analyzed of which 34 displayed significantly different levels of immunoreactivity in both replicates (Table 1). The spots with decreased phosphorylation (on the left) represent potential STK16 substrates whereas the spots with enhanced phosphorylation (on the right) likely represent other members of a phosphoprotein network that reciprocally regulate constitutive secretion (Fig. 3B).

**Table 1:** Many spots displayed significant changes in immunoreactivity on blots from E202A expressing cells.

Spot (#)	MW (kDa)	Fold-change	Spot (#)	MW (kDa)	Fold-change
1	n/d	**−3.3**	2	n/d	**13.1**
4	201.8	**−4.3**	16	164.1	**5.1**
25	157.5	**−2.4**	78	87.5	**3.2**
32	145.0	**−1.9**	95	77.5	**3.7**
52	120.9	**−3.0**	141	58.3	**3.8**
57	117.9	**−3.2**	165	53.4	**5.0**
73	91.0	**−2.4**	169	53.3	**4.3**
81	85.1	**−3.7**	184	50.1	**3.2**
92	78.7	**−5.5**	188	50.1	**3.1**
101	75.8	**−2.8**	201	47.5	**3.6**
112	71.5	**−2.6**	202	47.5	**4.4**
117	69.4	**−2.1**	216	44.7	**3.5**
123	65.1	**−2.4**	236	40.4	**4.7**
127	62.6	**−3.1**	239	39.6	**3.0**
172	52.3	**−4.0**	241	39.5	**4.2**
247	37.1	**−2.1**	243	39.3	**1.8**
276	27.5	**−8.5**	266	31.5	**3.7**

The overlapping spots shown in Fig. 3A were analyzed by densitometry (see Materials and Methods). From the 282 spots analyzed, 34 displayed altered immunoreactivity in E202A expressing cells. The spot numbers analyzed (see Fig. 3B) and apparent molecular weights for each are indicated. The fold-change in immunoreactivity is also indicated. The values on the left are those spots with decreased immunoreactivity whereas those on the right, had increased levels. Values represent averages from the technical duplicates.

To confirm the densitometry results, we visually examined enlarged regions of the immunoblot pairs from the duplicate immunoblots. Examples of these regions that showed reproducibly decreased or increased immunoreactivity are shown (Fig. 3C). Of the 34 spots examined, 30 spots (16 with decreased and 14 with increased) from both sets of immunoblots displayed equivalent changes in immunoreactivity and were selected for LC-MS/MS identification. In the end, 98 proteins were positively identified. Table S1 provides a complete list of the proteins with their isoelectric points, molecular weights, accession numbers and Mascot scores. The upper half of Table S1 includes those proteins with decreased immunoreactivity (possible STK16 substrates) while the bottom lists those proteins with enhanced immunoreactivity (possible reciprocal regulators). After discarding adenovirus-associated proteins and duplicate hits, we identified 28 proteins with decreased immunoreactivity and 40 proteins with enhanced immunoreactivity.

To better select possible regulators of constitutive secretion, we grouped the 68 proteins by subcellular location and general function. Table S2 lists those proteins with decreased phosphorylation while Table S3 lists those with increased phosphorylation. Although the LC-MS/MS approach used in these studies could not detect individual phosphorylated residues, the majority of these proteins (as indicated) have been previously identified as serine/threonine phosphorylated proteins either directly or by phospho-proteomic analysis validating our screen. The majority of the proteins identified are cytoplasmically-oriented thereby accessible to STK16 kinase activity further validating the results of the screen. Our bias during this initial screen was to select proteins whose known functions implicate them as possible regulators of vesicle budding/docking and/or fusion and those that encode a potential STK16 phosphorylation consensus sequence.

### WDR1 is a phosphoprotein

**Figure 4:**
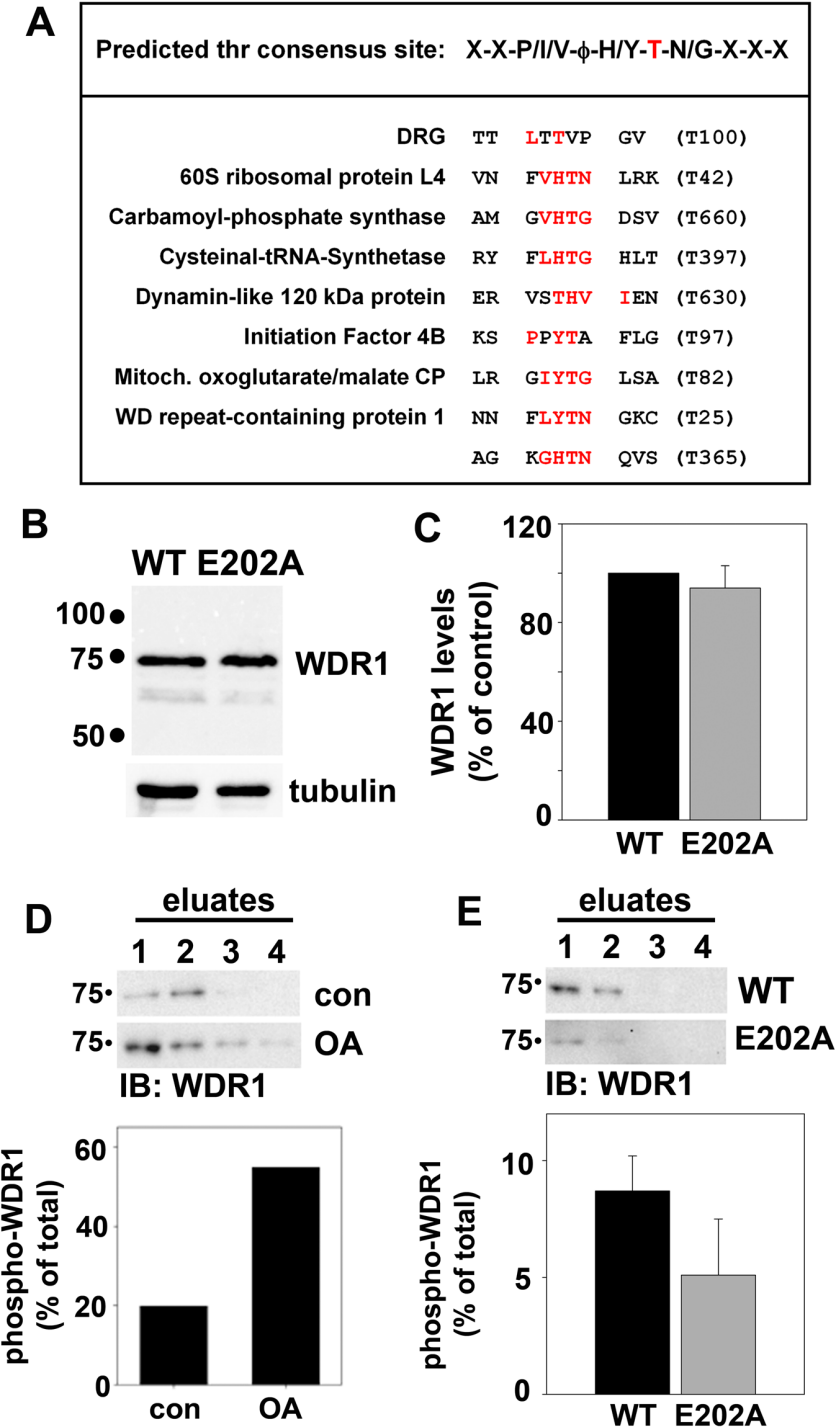
WDR1 is a phospho-protein. (**A**) The predicted threonine phosphorylation consensus sequence for STK16 is indicated at the top. The preferred threonine residue is highlighted in red and ϕ represents an aliphatic residue. For comparison, the known DRG phosphorylation site is shown. Sequence gazing revealed potential STK16 phosphorylation sites in seven of the proteins identified as possible STK16 substrates. WDR1 contains two such predicted phospho-threonine sites. The shared residues of the predicted sites with the consensus sequence are highlighted in red and the specific threonine indicated on the right in parentheses. (**B**) Total lysates from cells expressing wild type or E202A STK16 were immunoblotted for WDR1. Tubulin served as a loading control. Molecular weight standards are indicated on the left (in kDa). (**C**) The fold-change in WDR1 expression levels was determined by densitometric analysis of immunoreactive bands. Values were normalized to total tubulin levels and wild type expressing cells were set to 100%. Values represent the mean ± SEM determined from three independent experiments. (**D**) Lysates (~1 mg total protein) from cells treated with okadaic acid (OA) or (**E**) from cells expressing WT or E202A STK16 were applied to metal affinity columns to purify phospho-WDR1. The WDR1 in the eluate fractions represents the phosphorylated form. Molecular weight standards are indicated on the left (in kDa). From densitometric analysis of immunoreactive bands, the percent of WDR1 detected in the eluate fractions vs. total WDR1 loaded was determined and represents the percent phospho-WDR1. Values represent the average from two independent experiments in D and the average from three independent experiments ± SEM in E.

From peptide library screens, it was determined that STK16 has a strong preference for threonine over serine as a phospho-acceptor, and an optimal threonine phosphorylation sequence of X-X-P/I/V-ϕ-H/Y-T-N/G-X-X-X was identified^34^. We searched the sequences of the proteins with decreased immunoreactivity for this motif and found that 7 of the 28 proteins (25%) encoded such a sequence, and that WDR1 encoded two (Fig. 4A). For comparison, we have listed the threonine 100 phosphorylation site of developmentally regulated GTP-binding protein 1 (DRG1), a known STK16 binding partner and substrate that was used in the derivation of the consensus sequence along with the peptide library screens^34^. Because actin remodeling is required for hepatic secretion^35^, we chose WDR1, an actin-associated protein known to enhance cofilin actin disassembly activity (see Discussion), for further analysis. Importantly, endogenous WDR1 total protein levels were comparable in cells expressing wild type or E202A STK16 (Fig. 4B). When quantitated and normalized to total tubulin levels, WDR1 levels in E202A expressing cells were ~95% of control (Fig. 4C).

To determine if WDR1 is phosphorylated, we first purified phosphoproteins from WIF-B cells pre-treated with okadaic acid (a robust, pan-phosphatase inhibitor) using metal affinity chromatography optimized for phospo-protein retention (see Materials and Methods). Equal amounts of lysates (~1 mg total protein) from cells treated in the absence or presence of 100 nM okadaic acid were applied to columns and the flow through collected. After two washes, the columns were eluted in 4 ml of elution buffer collected in four fractions of 1 ml each. As shown in Fig. 4D, WDR1 was detected in the first three eluate fractions indicating that at steady state, and as predicted, WDR1 is a phosphoprotein. In eluates from okadaic acid-treated cells, increased levels of WDR1 were detected, peaking in the first two and detected in all four fractions, indicating enhanced associations with the column matrix and hence, increased WDR1 phosphorylation (Fig. 4D). When quantitated, we determined that WDR1 phosphorylation was enhanced in okadaic acid-treated cells by almost 3-fold (Fig. 4D).

To provide further evidence that WDR1 is a phosphoprotein and/or an STK16 substrate, we purified phosphoproteins from cells expressing either wild type or E202A STK16. As shown in Fig. 4E, lysates from cells expressing wild type STK16 showed similar elution profiles as control (uninfected) cells, peaking in the first two fractions with limited detection in the third. In contrast, WDR1 detection was significantly decreased in the first two eluate fractions from lysates expressing E202A indicating decreased phosphorylation (Fig. 4E). When quantitated, WDR1 phosphorylation levels in cells expressing wild type STK16 were ~2-fold greater than in E202A expressing cells (Fig. 4E). These results are consistent with the 2.8-fold decrease in phosphorylation observed on the 2D immunoblots (Table 1, spot 101). Although the values are not statistically significant, they are remarkably consistent given the many steps involved in the column purification and the numerous fractions collected and analyzed (see Materials and Methods). In fact, when examining individual experiments, the trend in the values was extremely reproducible. In all cases, less phospho-WDR1 was retained on the columns from cells expressing E202A when compared to those from wild type expressing cells (values ranged from as low as 10% of wild type values to 70%) which likely reflects differences in E202A infection efficiencies across experiments (see ref.^33^). These results indicate that WDR1 is a phosphoprotein.

**Figure 5:**
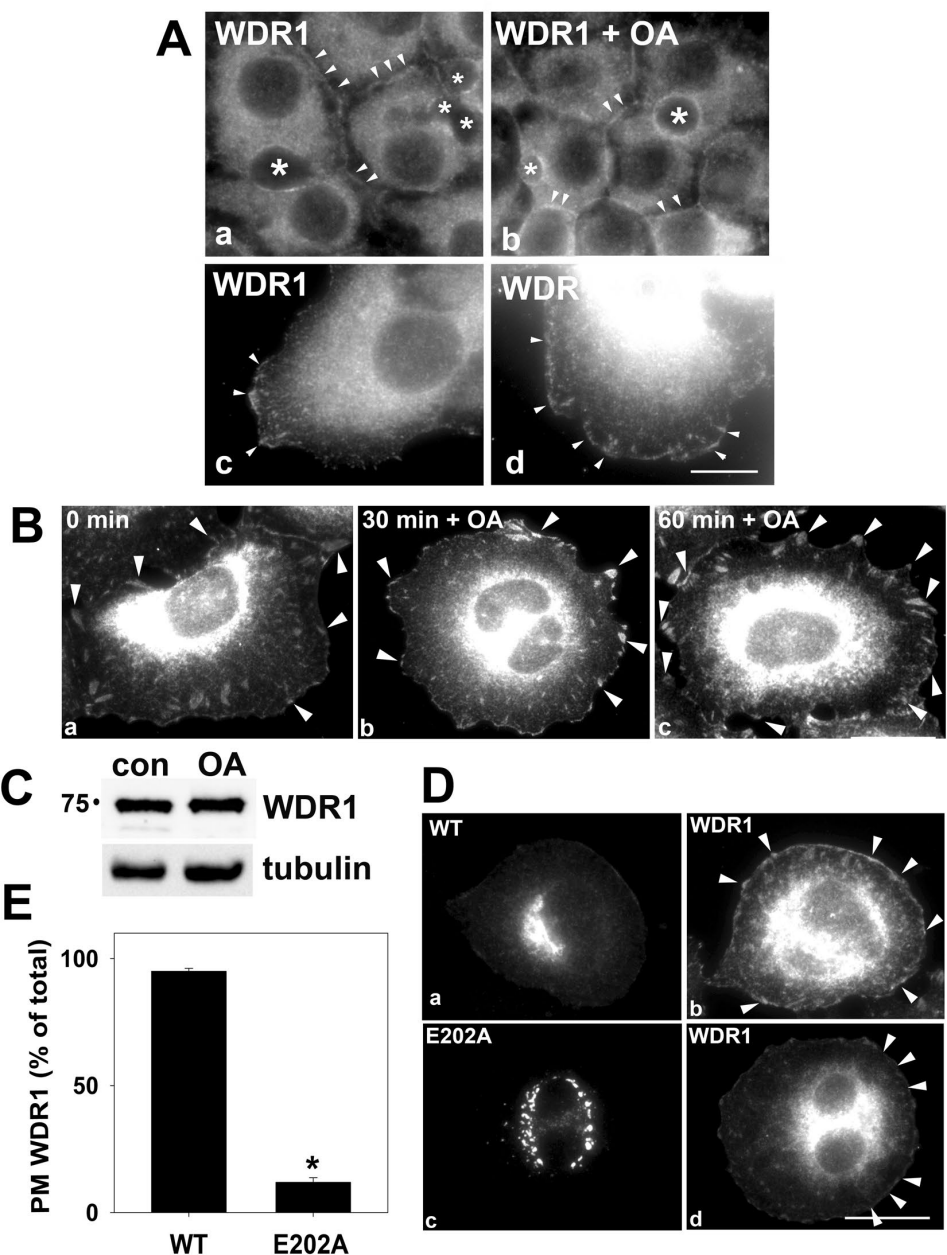
WDR1 distributions are phosphorylation-dependent. (**A**) WIF-B cells were treated in the absence or presence of 100 nM okadaic acid (OA) for 60 min and immunolabeled for WDR1. In a and b, WIF-B cells in the middle of the monolayer are shown whereas in c and d, cells at the edges of the monolayer were imaged. Arrowheads are pointing to WDR1 present at the apical and basolateral cell surfaces (**a**) or on plasma membrane patches (**c**). Arrowheads are marking the increased surface labeling of WDR1 in treated cells. Asterisks are marking bile canalicular spaces. Bar = 10 μm (**B**), Clone 9 cells were treated in the absence or presence of 100 nM okadaic acid (OA) for 0, 30 or 60 min and immunolabeled for WDR1. Arrowheads are marking the increased surface labeling of WDR1 in treated cells. Bar = 10 μm (**C)**, Lysates from Clone 9 cells treated in the absence or presence of 100 nM okadaic acid (OA) for 60 min were immunoblotted for WDR1. Tubulin served as the loading control. Molecular weight standards are indicated on the left (in kDa). (**D**), Clone 9 cells expressing wild type STK16 (WT) or E202A were immunolabeled for STK16 or WDR1 as indicated. Arrowheads are marking the surface associated WDR1 in cells expressing wild type, but not E202A-expressing cells. Bar = 10 μm **E**, Infected cells were scored for the presence or absence of cell surface WDR1 labeling and the percent of total infected cells with surface labeling was plotted. Values represent the mean ± SEM from three independent experiments. *p ≤ 0.001.

We next examined endogenous WDR1 steady state distributions in polarized WIF-B cells. As described for other polarized epithelial cells, WDR1 labeling was detected at or near the apical surface and in the cytoplasm in polarized WIF-B cells^36–38^ (Fig. 5A,a). We also detected WDR1 at the basolateral surface (marked with arrowheads) consistent with a role in basolateral secretion. We also noticed that WDR1 was detected in membrane-associated patches in cells at the edge of the monolayer (Fig. 5A,c; the images were intentionally overexposed to better visualize the surface labeling). If WDR1 serves as a constitutively phosphorylated protein in constitutive secretion, a simple prediction is that okadaic acid treatment should lead to altered WDR1 actin association at the plasma membrane. Consistent with this prediction, increased WDR1 labeling was detected at both the apical and basolateral membranes in cells treated with okadaic acid (Fig. 5A,b). In WIF-B cells at the edge of the monolayer, enhanced WDR1 membrane-association was also observed in okadaic acid-treated cells with respect to both staining intensity and numbers of surface-associated puncta (Fig. 5A,d, marked with arrowheads).

We also examined WDR1 distributions in the non-polarized, hepatoma-derived Clone 9 cells for ease of visualization and manipulation. As observed for other cell types and in WIF-B cells at the edge of the monolayer, endogenous WDR1 distributed to intracellular structures, the cytoplasm and at discrete patches at the plasma membrane (Fig. 5B,a). Also as observed for WIF-B cells at the edge of the monolayer, okadaic acid treatment led to enhanced WDR1 membrane association. Arrowheads are marking enlarged and more numerous WDR1-positive surface patches after 30 or 60 min of treatment (Fig. 5B,b,c). Importantly, total WDR1 protein levels did not change in okadaic acid-treated cells (Fig. 5C).

If WDR1 serves as a constitutively phosphorylated protein in response to STK16 activity, another simple prediction is that expression of wild type STK16 should lead to enhanced WDR1 surface labeling while expression of the dominant negative, kinase-dead E202A STK16 should decrease it. As we and others have shown previously, wild type STK16 distributed mainly to the Golgi whereas the kinase-dead E202A distributed to intracellular puncta (that have not yet been identified) [3] (Fig. 5D,a,c, respectively). As predicted, enhanced numbers of WDR1-positive surface patches were observed in the STK16 expressing cells to a similar extent as observed for okadaic acid-treated cells (compare Fig. 5D,b with Fig. 5B,c). In contrast, WDR1 surface labeling was nearly completely abolished in E202A-expressing cells (Fig. 5D,d). When cells were scored for WDR1 surface labeling, a striking loss of membrane staining was observed with only 11.9 ± 1.9% (p ≤ 0.001) of E202A-expressing cells positive for WDR1 surface patches (Fig. 5E) further implicating WDR1 as a phospho-protein.

### Constitutive basolateral secretion is regulated by WDR1 and actin dynamics

**Figure 6:**
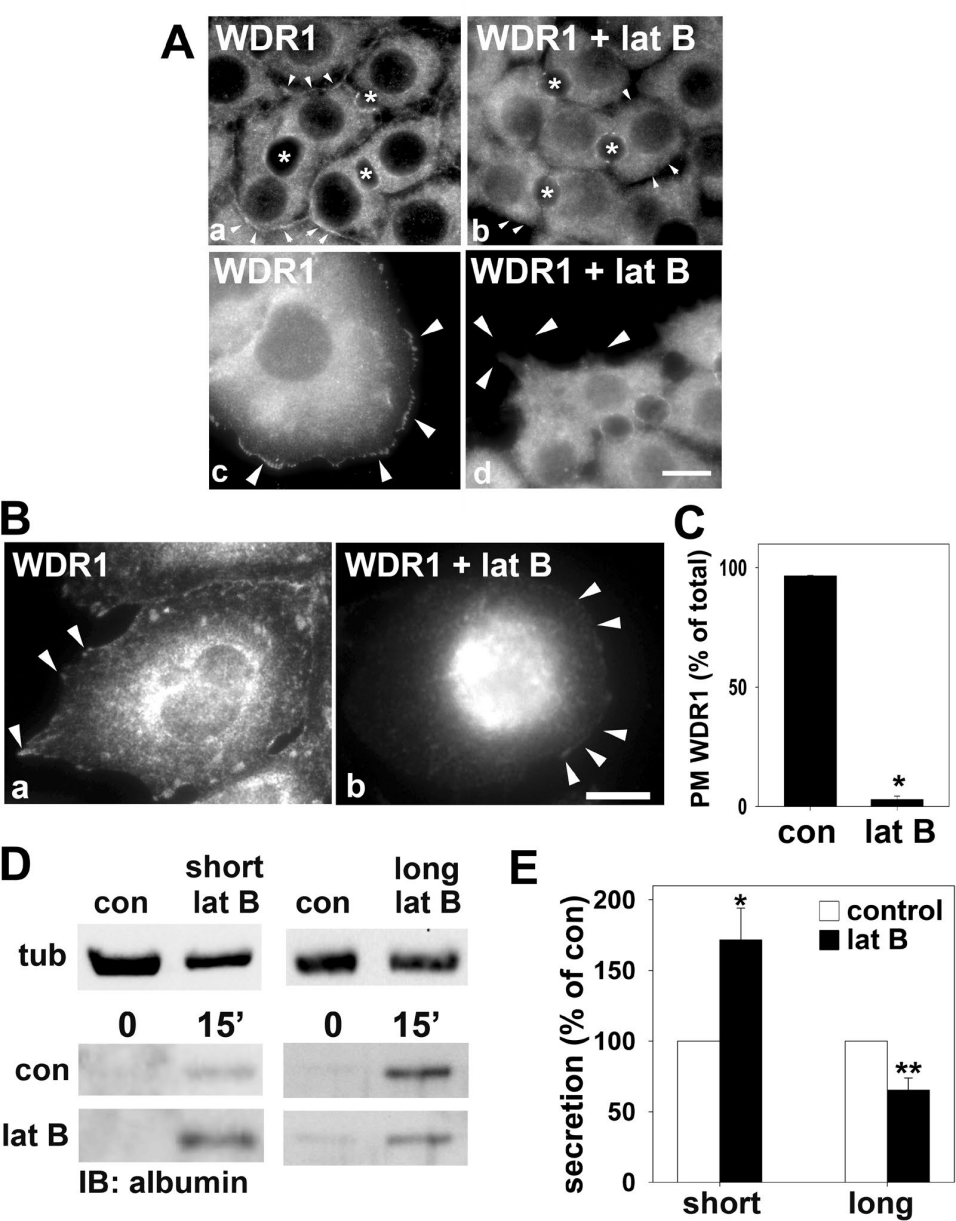
Basolateral secretion and WDR1 distributions are actin dependent. (**A**) WIF-B cells were treated in the absence or presence of 5 μM latrunculin B (lat B) for 60 min and immunolabeled for WDR1. In a and b, WIF-B cells in the middle of the monolayer are shown whereas in c and d, cells at the edges of the monolayer were imaged. Arrowheads are pointing to the actin-associated WDR1 present at the basolateral cell surface (a) or on plasma membrane patches (c). In panels b and d, arrowheads are marking the cell membrane lacking WDR1 labeling. Asterisks are marking the bile canalicular spaces. Bar = 10 μm (**B**), Clone 9 cells were treated in the absence or presence of 5 μM latrunculin B (lat B) for 30 min and immunolabeled for WDR1. Arrowheads are pointing to the actin-associated WDR1 present at the cell surface in panel a. Arrowheads in panel b are marking the cell periphery with no detectable WDR1. Bar = 10 μm (**C**), Cells were scored for the presence or absence of cell surface WDR labeling and the percent of total cells with surface labeling was plotted. Values represent the mean ± SEM from three independent experiments. *p ≤ 0.001 (**D**), WIF-B cells were pretreated with 10 μM latrunculin B (lat b) for 30 (short) or 60 min (long) at 37 °C. Cells were rinsed five times with prewarmed serum-free medium and then reincubated in serum-free medium. At 0 and 15 min after reincubation, aliquots of media were collected and analyzed for albumin secretion by immunoblotting (bottom panels). Whole cell extracts harvested after the secretion assay was performed were harvested and immunoblotted for tubulin (tub) to serve as a loading control (upper panels). (**D)** The relative levels of albumin secreted into the medium was calculated from densitometric analysis of immunoreactive bands on blots as shown in (**A**). Control values were set to 100%. Values are expressed as the mean ± SEM from three independent experiments. *p ≤ 0.029, **p ≤ 0.015.

To confirm WDR1 as a regulator of actin remodeling at sites of vesicle docking and fusion in hepatic cells, we next examined its distributions in cells treated with the actin depolymerizing agent, latrunculin B. As described above, WDR1 labeling was detected at or near the apical and basolateral surfaces and in the cytoplasm of polarized WIF-B cells (Fig. 6A,a). In contrast, WDR1 labeling at the membrane domains was lost in latrunculin B- (Fig. 6A,b and Fig. S1A,b) or cytochalasin D-treated cells (Fig. S1B,d) with a reciprocal increase in cytoplasmic labeling confirming that WDR1 distributions are actin-dependent in WIF-B cells. Similarly, the WDR1-positive membrane patches were lost in latrunculin B- (Fig. 6A,d, Fig. S1A,d or cytochalasin-treated cells present at the edge of the monolayer (Fig. S1B,e). A similar loss of the discrete WDR1 surface labeling was lost in latrunculin B- (Fig. 6B,b) or cytochalasin D-treated (Fig. S1B,f) Clone 9 cells with a reciprocal increase in cytoplasmic staining. When cells were scored for WDR1 surface labeling, nearly 100% (96.6 ± 0.3%) of control cells displayed WDR1-positive surface patches that were virtually all lost (2.9 ± 1.4%, p ≤ 0.001) in latrunculin B-treated cells (Fig. 6C). Importantly, surface labeling of WDR1 reappeared after latrunculin-B washout in both polarized and nonpolarized cells (Fig. S1A,c,f).

To confirm that basolateral secretion requires actin remodeling in WIF-B cells as seen for other polarized epithelial cells (see Discussion), we monitored albumin release in cells pretreated with latrunculin B for short times (to minimally disrupt the cortical actin network) *vs*. longer times (to more fully depolymerize the network). As observed for other epithelial cell types, shorter pretreatment reproducibly enhanced albumin secretion to almost twice that as observed in control (p ≤ 0.029), whereas the longer pretreatment reproducibly impaired release by ~40% (p ≤ 0.015) (Fig. 6D,E). The somewhat modest impairment after the longer preincubation likely reflects incomplete actin disassembly.

**Figure 7:**
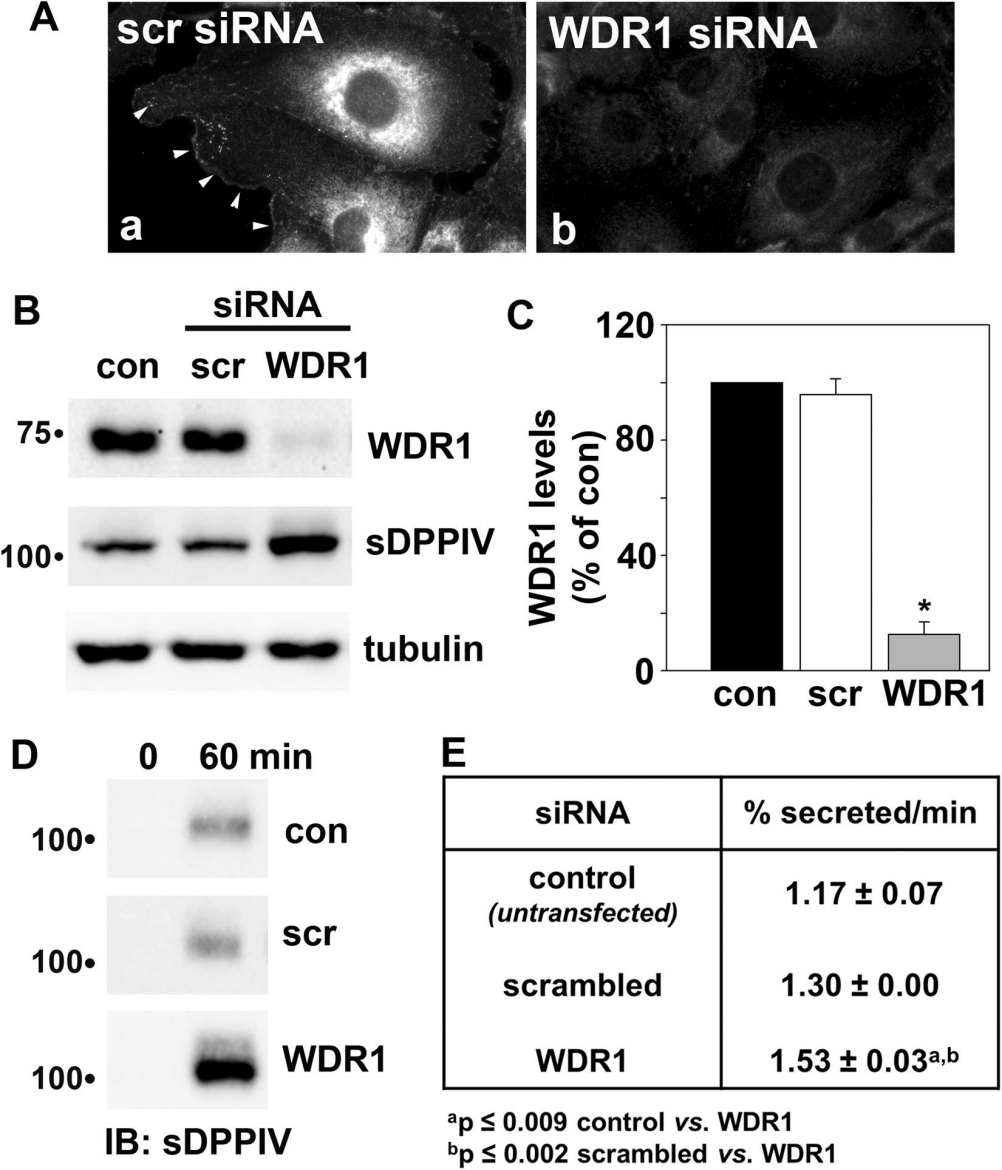
WDR1 knockdown enhances secretion. (**A**) Clone 9 cells were transfected with scrambled (scr) (panel a) or WDR1-specific siRNA duplexes (panel b), incubated for 48 h and immunolabeled for WDR1. Arrowheads are marking WDR1-positive cell surface patches in panel a. Note the considerable decrease in labeling in panel b. (**B**) Clone 9 cells were transfected with scrambled (scr) or WDR1-specific siRNA duplexes and incubated for 24 h. The control and transfected cells were infected with recombinant adenoviruses encoding V5-tagged sDPPIV and incubated an additional 24 h. Total cell lysates were immunoblotted for WDR1, sDPPIV and tubulin as indicated. Molecular weight standards are indicated on the left (in kDa). (**C**) The relative levels of WDR1 were calculated from densitometric analysis of immunoreactive bands on blots as shown in (**B**). Control values were set to 100%. Values are expressed as the mean ± SEM from three independent experiments. *p ≤ 0.001. (**D)** sDPPIV-expressing cells were rinsed five times with prewarmed serum-free medium and then reincubated in serum-free medium. At 0 and 60 min after reincubation, aliquots of media were collected and analyzed for sDPPIV secretion by immunoblotting with anti-V5 antibodies. (**E**) The percent sDPPIV secreted relative to the total expressed (**B**) was calculated from densitometric analysis of immunoreactive bands on blots as shown in (**D**) and the rate calculated. Values are expressed as the mean ± SEM from at least three independent experiments.

Finally, we examined a role for WDR1 in secretion by monitoring the release of a soluble form of dipeptidyl peptidase IV (sDPPIV), a well-characterized, constitutively secreted protein^39^, in cells knocked down for WDR1 expression. As shown in Fig. 7A,B, WDR1 expression was considerably knocked down morphologically and biochemically, respectively, after transfection with a pool of three WDR1-specific siRNA duplexes for 48 h. No change in WDR1 expression was observed in non-transfected cells or in cells transfected with scrambled duplexes (Fig. 7A,B). When quantitated, almost 90% knock down was observed in cells transfected with WDR1 siRNA (Fig. 7C). Similar levels of sDPPIV were expressed in control and WDR1 cells transfected with scrambled siRNA duplexes. However, a slight, but reproducible, increase in sDPPIV levels was observed in WDR1 knockdown cells (Fig. 7B). Tubulin served as the loading control.

When secretion was monitored in the same sets of cells as shown in Fig. 7B, we found that similar levels of sDPPIV were secreted after 60 min in both control cells and those transfected with scrambled siRNA. In contrast, much higher levels were detected in the media from cells knocked down for WDR1 expression (Fig. 7D). To take into account the increased sDPPIV expression in WDR1 knock down cells, we calculated the rates of secretion as the percent sDPPIV secreted of the total expressed per minute. A modest (~25%), but highly significant increase in secretion (p ≤ 0.009) was observed in WDR1 knock down cells (Fig. 7E). When compared to cells transfected with scrambled siRNA duplexes, the differences in secretion rates were found to be even more significant (p ≤ 0.002). Importantly, there was no significant difference observed in rates between control cells and those treated with scrambled siRNA. Together these data suggest that the WDR1-induced alterations in actin dynamics modulate secretion rates at sites of vesicle docking and fusion in hepatic cells.

## Discussion

A proteomics approach was used to identify either potential STK16 substrates or other regulators of constitutive secretion. In all, we identified 28 proteins that displayed decreased immunoreactivity in cells expressing the kinase-dead version of STK16 (E202A) that represent possible substrates. We also identified 40 proteins with enhanced phosphorylation. Although they are not likely STK16 substrates, they may be important players in a cellular kinase/phosphatase network that reciprocally regulates constitutive secretion. The majority of the proteins identified were cytoplasmically-oriented thereby accessible to STK16 kinase activity validating the results of the screen. Although the LC-MS/MS used was not optimized to detect phosphorylation, the majority of the proteins have been identified as phosphorylated on serine and/or threonine residues either from individual studies or from large phospho-proteomics screens. Sequence gazing also revealed that 25% of the possible substrates encoded a preferred threonine STK16 phosphorylation consensus sequence^34^, further validating our screen. Based on the finding that the actin-associated WDR1 encodes two such consensus sequences and that actin remodeling is required for hepatic secretion^35^, we further confirmed that WDR1 is a phospho-protein that regulates secretion.

### WDR1 is a phosphoprotein

Our results indicate that WDR1 is a phosphorylated protein that regulates constitutive secretion. Although we have not directly tested whether WDR1 is an STK16 substrate, it is a likely candidate. Not only does WDR1 encode two threonine-based STK16 consensus sequences, its phosphorylation state reciprocally correlates with wild type or E202A STK16 expression. Similarly, enhanced WDR1 surface association is observed in cells expressing wild type STK16 or treated with okadaic acid whereas surface distributions are lost in cells expressing E202A. Even if WDR1 is not found to be a direct STK16 substrate, it is at minimum, a part of a kinase/phosphatase network that regulates constitutive secretion. Proteomics screens have identified WDR1 as a tyrosine-phosphorylated protein and recent studies have demonstrated that it is a specific substrate for eyes absent 3 (EYA3) phosphatase at tyrosine 238^40^. In cells expressing Y238F mutants, only a 30% reduction in WDR1 phosphorylation was observed indicating other WDR1 phosphorylation sites must be present. Interestingly, EYA3 also exhibits threonine phosphatase activity possibly implying that these other sites may include the threonine-based STK16 consensus sites. We are currently examining this compelling possibility.

### WDR1 regulates secretion

WDR1 (also known as actin-interacting protein 1; AIP1) was first identified as an actin binding protein from yeast two hybrid analysis^41,42^. This protein encodes conserved WD40 repeats within β-propellers that serve as binding sites for actin and cofilin^43^. Recent studies in nonpolarized cells have shown that upon binding, WDR1 greatly enhances cofilin’s severing activity of both actin filaments and branched polymers leading to rapid actin disassembly^44^. Such activity has placed WDR1 as an important regulator of many cell processes in nonpolarized cells including cell migration via enhanced lamellipodial dynamics, cytokinesis and chemotaxis^45^. In polarized cells, WDR1 has also been associated with a number of complex cellular processes. In intestinal cells, WDR1 is enriched at apical junctions, and when knocked down, tight junction assembly is impaired and the juxta-junctional actin cytoskeleton is disorganized and displays altered dynamics^37^. Similarly, in the developing *Drosophila* eye epithelium, WDR1 and cofilin are required for adherens junction remodeling^36^. More recently, WDR1 depletion in developing mouse epidermis was found to compromise planar cell polarity, and when knocked down with cofilin, a loss of cell adhesion, surface and planar cell polarity was observed^38^. Our results now place WDR1/cofilin-mediated-actin remodeling as an important regulatory feature of constitutive secretion. Although WDR1 function in secretion was not examined in the systems described above, it may contribute to the complex phenotypes observed. For example, does WDR1 knockdown lead to altered membrane delivery (either to the apical or basolateral domain) of key components required for tight or adherens junction assembly?

Hints for how local actin modeling is required for secretory vesicle docking and fusion come from studies using actin disrupting agents. As for most other polarized epithelial cells, the polarized hepatocyte lacks actin stress fibers. Rather, a dense cortical actin web lines both the apical and basolateral surfaces that secretory vesicles must traverse to dock and fuse with the membrane. Early studies in chromaffin cells and neutrophils that demonstrated that local and transient actin disassembly were required for exocytosis are consistent with this actin barrier function in exocytosis^46,47^. However, more thorough studies using wider concentration ranges of actin depolymerizing agents revealed that actin remodeling at the sites of vesicle docking and fusion is actually a two-step process^48,49^. Low concentrations of actin disrupting agents enhanced exocytosis (consistent with the barrier function), but treatment with higher concentrations (that support complete actin disassembly) led to impaired exocytosis^48,49^. Thus, local disassembly is likely required for vesicle access to the membrane, but some minimal actin cytoskeletal structure is required for vesicle fusion^48,49^. This conclusion is consistent with our findings that WDR1 (that promotes actin disassembly) and members of the rho family of GTPases and their effectors (that promote actin assembly) are required for vesicle docking and fusion (reviewed in^48,50^).

At present, we have few mechanistic details of how WDR1 functions in secretion. Our findings that WDR1 knockdown led to enhanced secretion implies that WDR1/cofilin-mediated actin disassembly is regulating the actin barrier function in exocytosis. Because okadaic acid treatment and STK16 overexpression led to enhanced plasma membrane association of WDR1 whereas E202A expression led to diminished detection at the surface, further suggests that phosphorylation is required for WDR1 function in secretion. However this is in direct contrast to observations from HeLa cells knocked down for STK16 expression or expressing kinase dead STK16 where F-actin levels were decreased^51^. Because both increased actin disassembly via enhanced WDR1/cofilin severing activity and decreased WDR1 phosphorylation are predicted in this context, the implication is that phosphorylation impairs WDR1 activity. These disparate results point to the complexity in the fine tuning of actin remodelling required for proper cell function within and among cell types. Because WDR1 mutations have been linked to a number of human blood disorders and the progression of many different cancer types^45^, understanding the mechanisms regulating local actin remodelling have potential therapeutic ramifications.

## Materials and Methods

### Reagents and Antibodies

F12 and F12 (Coon’s modification) medium, latrunculin B, anti-α-tubulin mouse monoclonal antibodies (DM1A, Cat. no. T9026, lot no. 052M4837) and HRP-conjugated secondary antibodies were purchased from Sigma-Aldrich (St. Louis, MO). Lactacystin and okadaic acid were purchased from Enzo (Farmingdale, NY). Phosphoseek™ Phosphoprotein Enrichment kits were purchased from BioVision (Milipitas, CA). Mouse monoclonal antibodies against the V5 epitope tag (clone: SV5-Pk1, cat. no. MCA1360GA) were from AbD Serotec (Raleigh, NC) and rabbit monoclonal antibodies against WDR1 (Cat. No. EPR8793) were from AbCam (San Francisco, CA) or Santa Cruz (Dallas, TX) (Cat. No. B10). Mouse monoclonal antibodies against phospho-threonine (Q7, Cat. no. 1018223) and phospho-serine (Q5, Cat. no. 1018234) residues were purchased from Qiagen (Valencia, CA). Fetal bovine serum (FBS) was from Gemini Bio-Products (Woodland, CA). Polyclonal rabbit antibodies against rat serum albumin were generously provided by Dr. Ann Hubbard (Johns Hopkins University School of Medicine, Baltimore, MD).

### Cell Culture

WIF-B cells were grown in a humidified 7% CO_2_ incubator at 37 °C as described^52^. Briefly, cells were grown in F12 medium (Coon’s modification), pH 7.0, supplemented with 5% FBS, 10 μM hypoxanthine, 40 nM aminoterpin and 1.6 μM thymidine. Clone 9 cells were grown at 37 °C in a 5% CO_2_ incubator in F12 medium supplemented with 10% newborn calf serum. WIF-B cells were seeded onto glass coverslips at 1.3 × 10^4^ cells/cm^2^, whereas Clone 9 cells were seeded onto coverslips in 6-well dishes at 0.5–1.0 × 10^6^ cells/well. Clone 9 cells were cultured for 1–2 days and WIF-B cells for 8–12 days until they reached maximal density and polarity.

### Virus Production and Infection

Recombinant adenoviruses encoding C-terminally, V5 epitope-tagged full-length and kinase-dead STK16 (E202A) were generated using the ViraPower Adenoviral Expression System (Life Technologies, Carlsbad CA) according to the manufacturer’s instructions and as we have described^33^. The full-length and kinase-dead STK16 constructs have been described and characterized^33,53^. The virus encoding the V5-tagged sDPPIV has been described previously^39^. WIF-B cells were infected with recombinant adenovirus particles for 60 min at 37 °C as described^39^. Complete medium was added to the cells and they were incubated an additional 16–24 h to allow for protein expression.

### Sample Preparation

WIF-B cells expressing wild type or E202A were washed in TBS containing 1 mM sodium vanadate (Sigma Aldrich) and a phosphatase inhibitor cocktail (Set 1, EMD Millipore, Billerica, MA) diluted according to the manufacturer. The cells were lysed in 1% TX-100 in Hepes buffer (150 mM NaCl, 25 mM Hepes, pH 7.4) containing protease inhibitors (2 µg/ml each of leupeptin, antipain, PMSF and benzamidine) and the vanadate/phosphatase inhibitor cocktail and incubated on ice for 30 min. The lysates were either mixed with Laemmli sample buffer directly^54^ or centrifuged for 20 min at 1,000 × g at 4 °C to prepare post-nuclear supernatants. Protein concentrations of the whole cell or cleared lysates were determined using Bio-Rad protein assay (Bio-Rad, Hercules, CA) according to manufacturer’s instructions. 20 μg of protein was loaded into each lane on 1D gels. Immediately before 2D SDS-PAGE analysis, the samples were diluted with a 1:1 mixture of SDS PAGE: urea sample buffers. 550 μg of protein were loaded per 2D gel.

### Immunoblotting for 1D Gels

Proteins were electrophoretically separated using SDS-PAGE and transferred to nitrocellulose. After blocking for 1 h with Blotto (TBS containing 5% milk (w/v) and 0.1% Tween-20) for 1 h at RT, the blots were incubated overnight with the antibodies against both phospho-serine and -threonine (both 1:4000) in TBS containing 0.1% Tween-20 at 4 °C. When immunoblotting for STK16 or sDPPIV (with anti-V5 antibodies), WDR1, albumin or for α-tubulin, PBS was used instead of TBS. HRP-conjugated secondary antibodies were used at 5 ng/ml and immunoreactivity detected by enhanced chemiluminescence (Thermo Scientific, Waltham, MA) and a ChemiDoc Touch Imager System (Bio-Rad).

### 2D Gel Electrophoresis and Immunoblotting

2D electrophoresis was performed by Kendrick Labs, Inc. (Madison, WI) using the carrier ampholine method of isoelectric focusing^55,56^. Isoelectric focusing was performed in a 3.3 mm inner diameter glass tube using 2% pH 3–10 Isodalt Servalytes (Serva, Heidelberg, Germany) for 20,000 volt-hrs. The tube gel pH gradient plot was determined with a surface pH electrode. After equilibration for 10 min in buffer “0” (10% glycerol, 50 mM DTT, 2.3% SDS and 0.0625 M Tris, pH 6.8), each tube gel was sealed to the top of a stacking gel overlaying a 10% acrylamide slab gel (20 cm × 22 cm, 1.0 mm thick). The gels were dried between sheets of cellophane paper with the acid edge to the left. Duplicate gels were placed in transfer buffer (10 mM Caps, pH 11 containing 10% methanol) and transblotted onto PVDF.

The blots were stained with Coomassie Brilliant Blue R-250, desktop scanned, blocked for two hours in 5% BSA in Tween-20 in TBS (TTBS) and rinsed in TTBS. The blots were incubated with anti-phospho-serine and -threonine antibodies (both 1:25,000 in 2% BSA TTBS) overnight. HRP-conjugated secondary antibodies were used and immunoreactivity detected with enhanced chemiluminescence (Lumigen, Southfield, MI). To determine the fold change in phosphorylation in proteins from E202A expressing cells, the density of individual spots on both the gels and immunoblots were determined by scanning with a laser densitometer (Model PDSI, Molecular Dynamics Inc, Sunnyvale, CA) that was checked for linearity with a calibrated Neutral Density Filter Set (Melles Griot, Irvine, CA). The images were analyzed using Progenesis Same Spots software (version 4.5, 2011, TotalLab, UK) and Progenesis PG240 software (version 2006, TotalLab, UK). The level of each of the selected, fully-resolved immunoreactive spots was normalized to the relative protein level of its corresponding spot in the gel. Fold-increase in phosphorylation was calculated by comparing the wild type ratios to those from the E202A expressing samples. Values represent averages from the technical duplicates.

### Protein Digestion, Peptide Extraction and LC-MS/MS

Mass spectrometry was performed by the Department of Chemistry and Biomolecular Sciences at Clarkson University (Potsdam, NY). Selected proteins were excised, and the gel pieces processed according to published protocols^57–59^. The peptide mixtures were analyzed by reversed phase liquid chromatography (LC) and MS (LC-MS/MS) using a NanoAcuity UPLC (Micromass/Waters, Milford, MA) coupled to a Q-TOF Ultima API MS (Micromass/Waters, Milford, MA), according to published procedures^57,60–62^.

### Protein Identification

The raw data were processed using ProteinLynx Global Server (PLGS, version 2.4) software as described^60^ using the following parameters: background subtraction of polynomial order 5 adaptive with a threshold of 30%, two smoothings with a window of three channels in Savitzky-Golay mode and centroid calculation of the top 80% of peaks based on a minimum peak width of 4 channels at half height. The resulting pkl files were submitted for database search and protein identification to the public Mascot database search (www.matrixscience.com, Matrix Science, London, UK) using the following parameters: databases from NCBI, parent mass error of 1.3 Da, product ion error of 0.8 Da, enzyme used: trypsin. Up to one missed tryptic cleavage was allowed and cysteine propionamidation and methionine oxidation were considered. To identify false negatives, the following parameters were used: different organism databases, narrower error windows for the parent mass error (1.2 and then 0.2 Da) or the product ion error (0.6 Da) w, and up to two missed tryptic cleavage sites were allowed. The pkl files were also searched against an in-house PLGS database version 2.4 (www.waters.com) using similar parameters. To eliminate false positives, the MS/MS spectra of the proteins identified by only one peptide or with a Mascot score lower than 25 were verified.

### Immunofluorescence Microscopy

Control and infected cells were treated in the absence or presence of 5 μM latrunculin B or with 100 nM okadaic acid for up to 60 min at 37 °C in complete serum free medium, fixed on ice with chilled PBS containing 4% paraformaldehyde for 1 min and permeabilized with ice-cold methanol for 10 min. Cells were processed for indirect immunofluorescence as previously described^63^. Alexa 488- or 568-conjugated secondary antibodies were used at 3–5 µg/ml. Labeled cells were visualized at RT by epifluorescence with an Olympus BX60 Fluorescence Microscope (OPELCO, Dulles, VA) using an UPlanFl 60 × /NA 1.3, phase 1, oil immersion objective. Images were taken with an HQ2 CoolSnap digital camera (Roper Scientific, Germany) and Metamorph Imaging software (Molecular Devices, Sunny Vale, CA). Adobe Photoshop (Adobe Systems Inc, Mountain View, CA) was used to process images and to compile figures.

### Phospho-WDR1 purification

We purchased PhosphoSeek™ metal affinity chromatography kits from BioVision (Milpitas, CA) and purified phosphoproteins according to the manufacturer’s instruction. Cells expressing either wild type or E202A were scraped in TBS and recovered by centrifugation at 3,000 rpm for 5 min. Cells were washed three times with TBS, pelleted and resuspended in 2–3 volumes of the lysis buffer provided in the kit. Proteins were extracted by repeated freeze/thaw cycles and lysates cleared by centrifugation. Total protein was determined using Bio-Rad protein assay (Bio-Rad, Hercules, CA) according to the manufacturer’s instructions and concentrations adjusted to 10 mg/ml. Approximately 1 mg of total protein was mixed with 1.9 ml system buffer and applied to a pre-equilibrated column at RT. The flow through was collected and reapplied to the column three times to enrich for phosphoproteins. The last 2 ml of flow through were collected and saved in 1 ml fractions. The column was washed with 5 ml of the least stringent wash buffer provided and the first 2 ml was collected in fractions of 1 ml each. The column was washed again with 5 ml of the buffer provided and then with 10 ml of distilled water. The bound phosphoproteins were eluted with 4 ml of the elution buffer provided and collected into four fractions of 1 ml each. All fractions were were immunoblotted for WDR1. The total relative amount of protein was calculated after adjusting for volumes across all fractions. The amount recovered in the four eluates pooled together was determined relative to the total protein levels recovered in all fractions and plotted as a percent of total protein.

### WDR1 knockdown

Clone 9 cells were transfected with 100 nM universal scrambled negative control siRNA duplexes or a mixture of three rat WDR1-specific siRNA duplexes (100 nM each) (Origen, Rockville, MD) using Lipofectamine® RNAiMAX (Thermo-Fisher) according to the manufacturer’s instructions. Cells were incubated 48 h at 37 °C to allow WDR1 knockdown.

### Secretion assays

WIF-B cells in complete serum free medium were pretreated with 10 μM latrunculin B for 30 (short treatment) or 60 min (long treatment) at 37 °C. Cells were rinsed five times with prewarmed serum-free medium and then reincubated in serum-free medium. At 0 and 15 min after reincubation, aliquots of media were collected and analyzed for albumin secretion by immunoblotting. The cell lysates were collected by solubilization directly into SDS-PAGE sample buffer. Samples were processed for immunoblotting and densitometric analysis of immunoreactive bands.

Clone 9 cells were transfected with scrambled or WDR1-specific siRNA duplexes as described above and incubated for 24 h. The control and transfected cells were infected with recombinant adenoviruses encoding V5-tagged sDPPIV and incubated an additional 24 h. Cells were rinsed five times with prewarmed serum-free medium and then reincubated in serum-free medium. At 0 and 60 min after reincubation, aliquots of media were collected and analyzed for sDPPIV secretion by immunoblotting with anti-V5 antibodies. The cell lysates were collected by solubilization directly into SDS-PAGE sample buffer. Samples were processed for immunoblotting and densitometric analysis of immunoreactive bands.

### Densitometry and Statistical Analysis

Densitometric comparison of immunoreactive bands was performed using ImageJ software (National Institutes of Health). In general, images were inverted and averaged pixel intensity x area for each band was determined, blank-corrected and normalized to an appropriate loading control (see legends for details). Results were expressed as the mean ± SEM from at least three independent experiments. Comparisons between experimental groups were made using the Student’s t test for paired data. P values ≤ 0.05 were considered significant.

## Electronic supplementary material

Supplementary information

## Data Availability

All data generated or analysed during this study are included in this published article and its Supplementary Information files.
