# Supplementary material for: A Serine/Threonine Kinase 16-Based Phospho-Proteomics Screen Identifies WD Repeat Protein-1 As A Regulator Of Constitutive Secretion
Source: Sci Rep. 2018 Aug 29;8:13049. doi: 10.1038/s41598-018-31426-1 (PMC6115458; doi:10.1038/s41598-018-31426-1)
Supplement: Supplementary file 1 — Supplementary information [file 41598_2018_31426_MOESM1_ESM.pdf]

Supplementary Information

A SERINE/THREONINE KINASE 16-BASED PHOSPHO-PROTEOMICS SCREEN IDENTIFIES WD REPEAT PROTEIN-1 AS A REGULATOR OF CONSTITUTIVE SECRETION

Alfonso López-Coral*, Anneliese C. Striz* and Pamela L. Tuma

From Department of Biology, The Catholic University of America, Washington DC, 20064

***** These authors contributed equally to the manuscript

Address correspondence to: Pamela L. Tuma, PhD, Department of Biology, The Catholic University of America, Washington, DC 20064, ph: 202-319-6681; Fax: 202-319-5721;

Email: tuma@cua.edu

Figure S1. López-Coral, Striz and Tuma

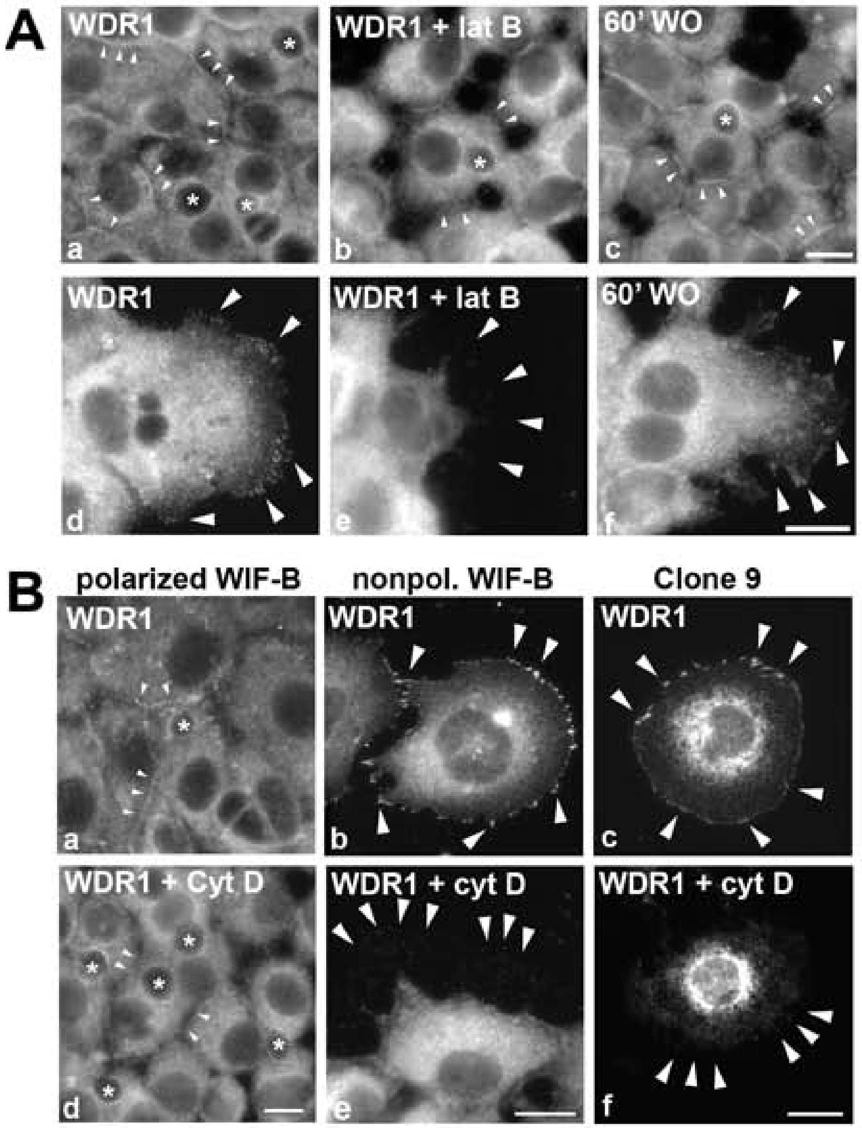

Figure S1. A, WIF-B cells were treated in the absence (a and d) or presence of 5 μM latrunculin B (lat B) for 60 min (b and e). The drug was then washed out for 60 min (60' WO) (c and d). All cells were immunolabeled for WDR1. In a-c, WIF-B cells in the middle of the monolayer are shown whereas in d-f, cells at the edges of the monolayer were imaged. Arrowheads are pointing to the actin-associated WDR1 present at the basolateral cell surface (a and c) or on plasma membrane patches (d and f). In panels b and e, arrowheads are marking the cell membrane lacking WDR1 labeling. Asterisks are marking the bile canalicular spaces. Bar = 10 μm **B,** Polarized WIF-B cells, non-polarized (nonpol.) WIF-B cells and Clone 9 cells were treated in the absence or presence of 10 μM cytochalasin D (cyt D) for 60 min and immunolabeled for WDR1. Arrowheads are pointing to the actin-associated WDR1 present at the basolateral cell surface (a) or on plasma membrane patches (b and c). In panels d-f, arrowheads are marking the cell periphery lacking WDR1 labeling. Asterisks are marking the bile canalicular spaces. Bar = 10 μm

Table S1. Numerous proteins display altered phosphorylation in E202A expressing cells.

Spot	Protein	NCBI Accession	App MW	Calc MW	Calc PI	Peptides Matched	Sequence Coverage (%)	Mascot Score
4	Vigilin isoform A	GI:4885409	142	141	6.43	9	8	233
25	Carbamoyl-phosphate synthase, mitochondrial	GI:8393186	166	165	6.33	4	3	160
52	Carbamoyl-phosphate synthase, mitochondrial	GI:124248512	166	165	6.48	3	2	103
57	Endoplasmin precursor	GI:210032365	93	93	4.72	9	10	480
67	Protein OS-9	GI:564359598	70	N/A	4.75	4	9	174
73	Carbamoyl-phosphate synthase	GI:21361331	165	166	6.30	7	6	162
	Dynamin-like 120 kDa protein, mitochondrial	GI:18860831	108	112	8.08	4	4	107
	Cysteinal-tRNA-Synthetase	GI:11191800	96	95	6.32	2	3	93
	Initiation Factor 4B	GI:288100	69	69	5.49	3	6	73
81	Poly(A) binding protein	GI:53754	71	69	9.45	12	21	420
	Mitochondrial long-chain enoyl-CoA hydratase/ 3-hydroxycyl-CoA dehydrogenase α-subunit	GI:510108	83	83	9.11	3	6	95
	Elongation factor 2	GI:4503483	96	96	6.41	2	2	65
92	Serum albumin	GI:30794280	72	69	5.82	37	53	1791
	Phosphoenolpyruvate carboxykinase	GI:38454296	70	69	6.09	21	44	1036
	WD repeat-containing protein 1	GI:62078997	66	67	6.15	11	23	638
	Ran-GTPase activating protein	GI:349501086	64	64	4.64	5	11	191
101	Phosphoenolpyruvate carboxykinase	GI:28454296	70	69	6.09	21	44	1036
	WD repeat-containing protein 1	GI:62078997	67	66	6.15	11	23	638
	Ran GTPase-activating protein 1	GI:349501086	64	64	4.64	5	11	191
112	hnRNP Kisoform 2	GI:13384620	51	51	5.31	22	33	686
117	Carbamoyl-phosphate synthase, mitochondrial	GI:21361331	166	166	6.33	7	6	284
	T-protein complex 1 subunit γ	GI:40018616	61	61	6.23	4	7	250
	Malic enzyme 1	GI:149018984	56	64	5.77	4	10	155
123	Heat shock protein 6	GI:51455	61	61	5.91	51	66	2121
	Chaperonin subunit 8	GI:149059760	55	60	5.15	13	21	537
172	Microsomal epoxide hydrolase	GI:205372	48	53	8.62	11	25	510
	60S ribosomal protein L4	GI:11968086	48	47	10.92	8	18	354
	Elongation factor 1α	GI:148694454	38	40	9.13	4	16	27
247	Malate dehydrogenase, cytoplasmic	GI:15100179	37	36	6.16	21	50	9181
263	Proteasome subunit, α type 3	GI:30584117	28	28	5.19	7	19	180
	Mitochondrial 2-oxoglutarate/malate carrier protein	GI:21312994	34	34	9.95	2	8	109
276	Peroxiredoxin-6	GI:16758348	25	25	5.64	8	54	630
	Glutathione S-transferase α-2	GI:58331251	26	26	8.89	3	13	144

	PREDICTED: NADH dehydrogenase flavoprotein 2, mitochondrial	GI:345329641	28	27	8.20	2	9	107
	Thioredoxin-dependent peroxide reductase, mitochondrial	GI:5802974	28	28	7.67	1	4	64
2	Carbamoyl-phosphate synthase, mitochondrial	GI:8393186	166	166	6.33	20	13	421
78	N-ethylmaleimide-sensitive factor	GI:467977	84	35	6.28	4	5	82
	Protein disulfide-isomerase	GI:16758712	73	73	4.99	5	10	82
	Non-muscle caldesmon	GI:227429	62	61	6.36	2	3	69
95	Transketolase	GI:149034221	72	68	7.54	11	25	558
	Dolichyl-diphosphooligosaccharide-protein glycosyltransferase subunit 1	GI:132560	68	69	6.05	6	11	273
	Calcium-binding mitochondrial carrier protein Aralar2	GI:392339831	75	74	8.86	9	11	257
	p68 RNA helicase	GI:51263	70	69	9.06	8	14	248
	Gry-rbp/hnRNP K	GI:3037013	70	70	8.68	2	4	164
	Very long-chain specific acyl-CoA dehydrogenase, mitochondrial precursor	GI:6978435	71	70	9.01	5	8	134
	Growth regulated nuclear 68 protein	GI:226021	68		8.95	7	12	244
	Aralar2	GI:6523256	75	74	8.87	7	8	174
	RNase L inhibitor	GI:987870	69	68	8.86	7	12	109
	DEAD (Asp-Glu-Ala-Asp) box polypeptide 17	GI:119580652	61	60	7.94	4	8	109
	70kDa peroxisomal membrane protein/ABCD3	GI:35553	76	70	9.47	1	2	90
	Dolichyl-diphosphooligosaccharide-protein glycosyltransferase subunit 1	GI:4506675	69	69	5.96	3	3	59
	Ribophorin I	GI:149036687	68	69	6.05	7	12	283
	RRM RNA binding protein NSAP1	GI:15986735	63	66	9.27	2	4	164
	ABC E1	GI:157823309	68	67	8.63	9	15	139
141	Serine hydroxymethyltransferase, mitochondrial	GI:564359582	56	56	8.46	32	54	1488
	UTP--glucose-1-phosphate uridylyltransferase	GI:67078526	57	57	7.18	15	30	516
	ATP synthase α subunit	GI:203055	59	59	9.22	8	21	385
	UDP-glucuronosyltransferase 1-1	GI:28849913	61	61	8.77	4	10	114
	Plasminogen activator inhibitor 1 RNA-binding protein	GI:524952264	45	45	8.31	8	24	113
	UDP-glucuronosyltransferase 1-6	GI:136726	61	61	8.91	2	6	72
	Serine hydroxymethyltransferase, mitochondrial	GI:19923315	57	57	8.76	33	49	1382
	UTP-glucose-1-phosphate uridylyltransferase isoform a	GI:48255966	58	57	8.16	17	32	444
	ATP synthase subunit a, mitochondrial	GI:4757810	48	60	9.16	8	21	332
	Polypyrimidine tract-binding protein 1 isoform a	GI:4506243	60	60	9.21	1	4	162
	CGI-55 protein/STARD10	GI:4929579	43	43	8.42	7	19	99

	ATP synthase subunit α, mitochondrial	GI:6680748	60	60	9.22	8	21	332
	Pyrimidine binding protein 1	GI:57002	57	57	9.14	3	8	186
	Protein RCC2	GI:507547646	57	57	9.02	3	7	115
169	Glutathione synthetase	GI:25742757	53	53	5.48	17	34	804
	Eukaryotic peptide chain release factor subunit 1	GI:4759034	49	49	5.51	10	20	175
	Phenylalanine-4-hydroxylase	GI:4557819	52	52	6.15	3	5	105
	Glutathione synthetase	GI:4504169	53	53	5.67	7	10	346
188	PREDICTED: γ-enolase isoform 2	GI:472389324	43	47	5.04	10	39	377
201	Phenylalanine-4-hydroxylase	GI:129975	52	52	5.76	19	44	835
	Mitochondrial-processing peptidase subunit β	GI:122065519	55	54	6.25	14	22	623
	Palmitoylated Serine/Threonine kinase	GI:4106342	35	34	6.05	3	10	138
202	Cytochrome b-c1 complex subunit 1, mitochondrial	GI:51948476	54	53	5.57	7	15	208
	Glutathione synthetase isoform a	GI:6680117	52	52	5.56	2	6	59
	γ-actin	GI:809561	41	42	5.56	11	23	394
	β-actin variant	GI:62897625	42	42	5.37	11	23	397
216	Ferrochelatase mitochondrial	GI:564393790	52	48	9.18	10	17	205
	RNA binding protein p45AUF1	GI:9588096	38	38	7.62	6	18	107
	DNA-binding protein	GI:181914	36	36	8.86	4	13	47
236	Phosphotriesterase-related protein	GI:61889077	40	39	6.40	11	36	620
	Ubiquitin fusion-degradation 1 like protein	GI:1654346	39	39	6.04	3	11	121
	N-acetylneuraminic acid synthase	GI:149045843	32	40	6.34	2	11	80
239	Aflatoxin B1 aldehyde reductase member 2	GI:19705537	38	40	6.27	16	37	458
	Aflatoxin aldehyde reductase	GI:2736256	37	40	6.23	9	21	210
	Serine dehydratase-like	GI:157820447	36	35	6.12	4	13	115
241	CGI-52 protein/STARD10	GI:4929573	41	33	8.54	10	29	551
	Transaldolase	GI:12002054	38	37	6.57	10	23	457
	LIM and SH3 domain protein 1	GI:14249130	30	30	6.61	6	27	341
	60S acidic ribosomal protein P0	GI:11693176	34	34	5.91	6	27	294
243	Aflatoxin B1 aldehyde reductase member 2	GI:19705537	38	41	6.27	23	53	881
	Aldose 1-epimerase	GI:56090564	38	38	6.18	6	23	256
	Farnesyl pyrophosphate synthase	GI:120478	41	48	5.29	1	4	100
266	Enoyl-Coa Hydratase, mitochondrial	GI: 17530977	32	32	8.39	3	16	103
	Phosphoglycerate mutase 1	GI:4505753	29	29	6.67	13	46	329

The isoelectric points, molecular weights, accession numbers and Mascot scores for each protein identified by mass spectrometry are listed. The upper half of the table includes those proteins with decreased immunoreactivity (possible STK16 substrates) while the bottom lists the proteins with enhanced immunoreactivity (possible reciprocal regulators).

TABLE S2. General functions of proteins with decreased phospho-immunoreactivity

Protein	General Function	Subcellular Location	Phospho-protein?	Refs.
60S ribosomal protein L4	Protein translation	cyt	ser	[1, 2]
Cysteinal-tRNA-synthetase	Protein translation	cyt	ser	[3, 4]
Elongation factor 1α	Protein translation	cyt	thr	[5, 6]
Elongation factor 2	Protein translation	cyt	ser/thr	[6]
Initiation factor 4B	Protein translation	cyt	ser/thr	[2, 4, 7]
Poly(A) binding protein	Protein translation	cyt	ser	[4, 8]
Serum albumin precursor	Blood pressure regulation (secretory protein)	ER/Golgi		
Chaperonin subunit 8	Molecular chaperone	cyt		
Heat shock protein 65	Molecular chaperone	mito	ser	
T-protein complex 1 subunit γ	Molecular chaperone	cyt	ser	[3, 7, 9]
Endoplasmin	ER chaperone/ERAD	ER	ser/thr	[2, 3, 7]
Protein OS-9	ERAD	ER		
Proteasome subunit, α type 3	Protein degradation	cyt	ser	[2-4, 9-11]
Glutathione S-transferase α-2	Oxidative stress	cyt		
Peroxiredoxin-6	Oxidative stress	cyt/lys	thr	[2, 4]
Thioredoxin-dependent peroxide reductase	Oxidative stress	mito	thr	[1, 2]
Microsomal epoxide hydrolase	Detoxification	ER		
γ-enolase isoform 2	Carbohydrate metabolism	cyt	ser/thr	[2]
Malate dehydrogenase	Carbohydrate metabolism	cyt	ser	[2, 3]
Mitochondrial 2 oxoglutarate-malate carrier protein	Carbohydrate metabolism	mito		
PEP carboxykinase	Carbohydrate metabolism	cyt	ser/thr	[3]
NADH dehydrogenase flavoprotein 2	Electron transport	mito		
Mitochondrial long-chain enoyl-CoA hydratase/3-hydroxyacyl-CoA dehydrogenase	Lipid metabolism	mito	ser/thr	[3]
Vigilin A (HDL binding protein)	Sterol metabolism	cyt	ser/thr	[2-4, 7, 9, 12]
Carbamoyl-phosphate synthase	AA metabolism; urea cycle	mito	ser	[3]
WD repeat-containing protein-1	Actin dynamics	cyt		
Ran-GAP	Nuclear transport	cyt/nuc	ser/thr	[13]
Dynamin-like 120 kDa protein	Mitochondrial fusion	mito		

The identified proteins with decreased phospho-serine/threonine immunoreactivity are grouped according to function. The subcellular location of each protein is indicated. Known serine- or threonine-phospho-proteins are also indicated and the references are provided. cyt, cytosol; mito, mitochondria; nuc, nucleus; Refs., references cited

TABLE S3. General functions of proteins with increased phospho-immunoreactivity.

Protein	General Function	Subcellular Location	Phospho-protein?	Refs.
p68 RNA helicase	Transcriptional regulation	nuc	ser	[2, 7]
DEAD (Asp-Glu-Ala-Asp) box polypeptide	Transcriptional regulation	nuc		
RNA binding protein p45AUF1	Transcriptional regulation	nuc		
Pyrimidine binding protein 1	Transcriptional regulation	cyt	ser/thr	[2, 4, 7, 14]
60S acidic ribosomal protein P0	Protein translation	cyt/nuc	ser	[12]
Eukaryotic peptide chain release factor subunit 1 isoform 1	Protein translation	cyt		
Protein with homology to EIF-4A	Protein translation	cyt/nuc	ser	[2, 4, 9]
Ferrochelatase	Porphyrin synthesis	mito		
Ribophorin I	Glycoprotein processing	ER		
Protein disulfide-isomerase A4	ER protein chaperone	ER		
Ubiquitin fusion-degradation 1 like protein	Protein degradation	cyt	ser	[2, 4, 7, 9]
Glutathione synthetase	Oxidative stress	cyt	ser	[3]
Aflatoxin B1 aldehyde reductase2	Detoxification	cyt	ser	[3]
UDP-glucuronosyltransferase 1-6	Detoxification	ER		
Aldose 1-epimerase	Carbohydrate metabolism	cyt		
N-acetylneuraminic acid synthase	Carbohydrate metabolism	cyt		
Citrate synthase	Carbohydrate metabolism	mito		
Phosphoglycerate mutase 1	Carbohydrate metabolism	cyt	ser	[2-4, 7, 9]
Phosphotriesterase-related protein	Carbohydrate metabolism	cyt		
Transaldolase	Carbohydrate metabolism	cyt	ser	[3]
Transketolase	Carbohydrate metabolism	cyt	ser/thr	[2, 4, 9]
UTP-glucose-1-phosphate uridylyltransferase	Carbohydrate metabolism	cyt	ser/thr	[2, 3, 7]
ATP synthase α subunit	Cellular respiration	mito	ser	[3, 7]
Cytochrome b-c1 complex subunit 1	Cellular respiration	mito		
70kDa peroxisomal membrane protein/ABCD3	Lipid metabolism (BA synthesis)			

Enoyl-Coa Hydratase	Lipid metabolism	mito	ser/thr	[3]
Farnesyl pyrophosphate synthase	Lipid metabolism	cyt		
Very long-chain specific acyl-CoA dehydrogenase	Lipid metabolism	mito		
CGI-52 protein/STARD10	Phospholipid transfer	cyt	ser	[3, 15]
Calcium-binding mitochondrial carrier protein Aralar2	AA metabolism	mito		
Phenylalanine-4-hydroxylase	AA metabolism	cyt	ser	[3, 16]
Serine hydroxymethyltransferase	AA metabolism	cyt		
γ-actin	Actin cytoskeleton	cyt		
β–actin variant	Actin cytoskeleton	cyt		
Caldesmon	Actin dynamics	cyt	ser	[2-4, 7, 8, 14]
LIM and SH3 domain protein 1	Actin dynamics	cyt	ser/thr	[2-4, 9, 14, 17]
Protein RCC2	Mitosis/rac GEF (?)	cyt	ser/thr	[4, 8, 9]
Mitochondrial-processing peptidase	Mitochondrial protein targeting	mito		
STK16	Constitutive secretion	cyt/nuc	ser/thr	[18]
NEM sensitive factor	Vesicle docking & fusion	cyt		

The identified proteins with increased phospho-serine/threonine immunoreactivity are grouped according to function. The subcellular location of each protein is indicated. Known serine- or threonine-phosphoproteins are also indicated and references are cited. BA, bile acid; cyto, cytosol; mito, mitochondria; NEM, N-ethylmaleimide; nuc, nucleus; Refs., references cited

REFERENCES

1 Daub, H., Olsen, J. V., Bairlein, M., Gnad, F., Oppermann, F. S., Korner, R., Greff, Z., Keri, G., Stemmann, O. and Mann, M. (2008) Kinase-selective enrichment enables quantitative phosphoproteomics of the kinome across the cell cycle. Mol Cell. **31**, 438-448

2 Olsen, J. V., Vermeulen, M., Santamaria, A., Kumar, C., Miller, M. L., Jensen, L. J., Gnad, F., Cox, J., Jensen, T. S., Nigg, E. A., Brunak, S. and Mann, M. (2010) Quantitative phosphoproteomics reveals widespread full phosphorylation site occupancy during mitosis. Sci Signal. **3**, ra3

3 Bian, Y., Song, C., Cheng, K., Dong, M., Wang, F., Huang, J., Sun, D., Wang, L., Ye, M. and Zou, H. (2013) An enzyme assisted RP-RPLC approach for in-depth analysis of human liver phosphoproteome. J Proteomics. **96**, 253-262

4 Dephoure, N., Zhou, C., Villen, J., Beausoleil, S. A., Bakalarski, C. E., Elledge, S. J. and Gygi, S. P. (2008) A quantitative atlas of mitotic phosphorylation. Proc Natl Acad Sci U S A. **105**, 10762-10767

5 Eckhardt, K., Troger, J., Reissmann, J., Katschinski, D. M., Wagner, K. F., Stengel, P., Paasch, U., Hunziker, P., Borter, E., Barth, S., Schlafli, P., Spielmann, P., Stiehl, D. P., Camenisch, G. and Wenger, R. H. (2007) Male germ cell expression of the PAS domain kinase PASKIN and its novel target eukaryotic translation elongation factor eEF1A1. Cell Physiol Biochem. **20**, 227-240

6 Hizli, A. A., Chi, Y., Swanger, J., Carter, J. H., Liao, Y., Welcker, M., Ryazanov, A. G. and Clurman, B. E. (2012) Phosphorylation of eukaryotic elongation factor 2 (eEF2) by cyclin A-cyclin-dependent kinase 2 regulates its inhibition by eEF2 kinase. Mol Cell Biol. **33**, 596-604

7 Rigbolt, K. T., Prokhorova, T. A., Akimov, V., Henningsen, J., Johansen, P. T., Kratchmarova, I., Kassem, M., Mann, M., Olsen, J. V. and Blagoev, B. (2011) System-wide temporal characterization of the proteome and phosphoproteome of human embryonic stem cell differentiation. Sci Signal. **4**, rs3

8 Olsen, J. V., Blagoev, B., Gnad, F., Macek, B., Kumar, C., Mortensen, P. and Mann, M. (2006) Global, in vivo, and site-specific phosphorylation dynamics in signaling networks. Cell. **127**, 635-648

9 Mayya, V., Lundgren, D. H., Hwang, S. I., Rezaul, K., Wu, L., Eng, J. K., Rodionov, V. and Han, D. K. (2009) Quantitative phosphoproteomic analysis of T cell receptor signaling reveals system-wide modulation of protein-protein interactions. Sci Signal. **2**, ra46

10 Han, G., Ye, M., Zhou, H., Jiang, X., Feng, S., Tian, R., Wan, D., Zou, H. and Gu, J. (2008) Large-scale phosphoproteome analysis of human liver tissue by enrichment and fractionation of phosphopeptides with strong anion exchange chromatography. Proteomics. **8**, 1346-1361

11 Wang, X., Chen, C. F., Baker, P. R., Chen, P. L., Kaiser, P. and Huang, L. (2007) Mass spectrometric characterization of the affinity-purified human 26S proteasome complex. Biochemistry. **46**, 3553-3565

12 Oppermann, F. S., Gnad, F., Olsen, J. V., Hornberger, R., Greff, Z., Keri, G., Mann, M. and Daub, H. (2009) Large-scale proteomics analysis of the human kinome. Mol Cell Proteomics. **8**, 1751-1764

13 Zhai, B., Villen, J., Beausoleil, S. A., Mintseris, J. and Gygi, S. P. (2008) Phosphoproteome analysis of Drosophila melanogaster embryos. J Proteome Res. **7**, 1675-1682

14 Yu, L. R., Zhu, Z., Chan, K. C., Issaq, H. J., Dimitrov, D. S. and Veenstra, T. D. (2007) Improved titanium dioxide enrichment of phosphopeptides from HeLa cells and high confident phosphopeptide identification by cross-validation of MS/MS and MS/MS/MS spectra. J Proteome Res. **6**, 4150-4162

15 Olayioye, M. A., Buchholz, M., Schmid, S., Schoffler, P., Hoffmann, P. and Pomorski, T. (2007) Phosphorylation of StarD10 on serine 284 by casein kinase II modulates its lipid transfer activity. J Biol Chem. **282**, 22492-22498

16 Miranda, F. F., Teigen, K., Thorolfsson, M., Svebak, R. M., Knappskog, P. M., Flatmark, T. and Martinez, A. (2002) Phosphorylation and mutations of Ser(16) in human phenylalanine hydroxylase. Kinetic and structural effects. J Biol Chem. **277**, 40937-40943

17 Zahedi, R. P., Lewandrowski, U., Wiesner, J., Wortelkamp, S., Moebius, J., Schutz, C., Walter, U., Gambaryan, S. and Sickmann, A. (2008) Phosphoproteome of resting human platelets. J Proteome Res. **7**, 526-534

18 Eswaran, J., Bernad, A., Ligos, J. M., Guinea, B., Debreczeni, J. E., Sobott, F., Parker, S. A., Najmanovich, R., Turk, B. E. and Knapp, S. (2008) Structure of the human protein kinase MPSK1 reveals an atypical activation loop architecture. Structure. **16**, 115-124

Full length Gels and Blots

- The 1D immunoblots shown in Figures 1B and 2B are full-length.
- The 2D CBB-blue stained gels shown in Figure 2C are full-length and the 2D immunoblots shown in Figures 2C, 3A and 3B are also all full-length.
- The cropped immunoblots shown in Figure 3C are from the immunoblots shown in Figure 2C.
- The full-length immunoblots that were cropped for the figures are provided below. The cropped regions are indicated with red boxes and brief legends are provided.

Figure 1A

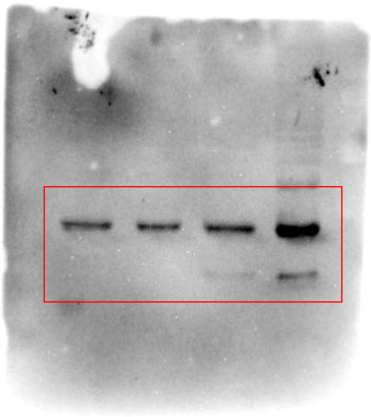

Top panel:
IB: anti-V5 (to detect tagged STk16)
STK16 WT ± lac/E202A right ± lac

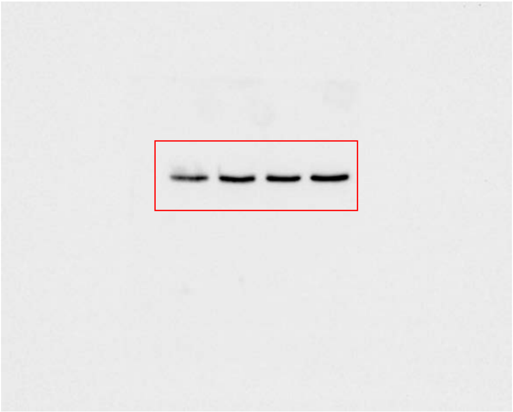

Bottom panel:
IB: anti-α-tubulin
STK16 WT ± lac/E202A right ± lac
(different blot with the same samples from above)

Figure 2A

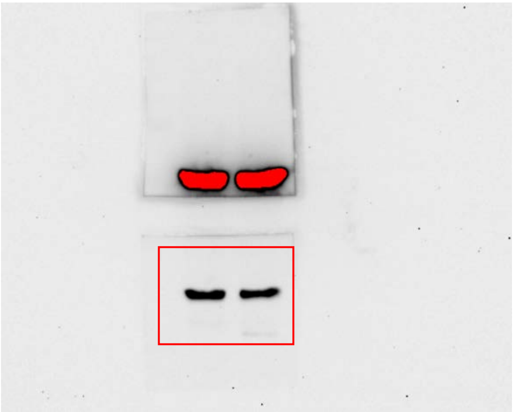

Top panel:
IB: anti-V5 (to detect tagged STK16)
WCE from cells expressing STK16 WT/E202A

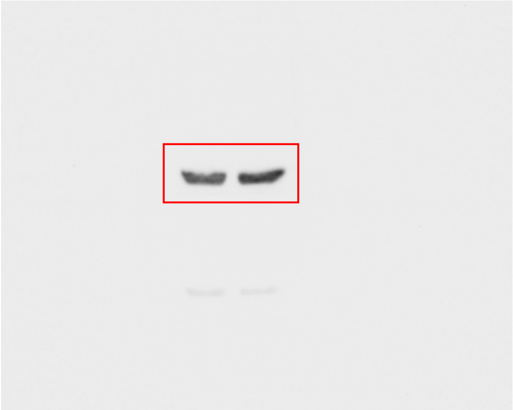

Bottom panel:
IB: anti-α-tubulin
WCE from cells expressing STK16 WT/E202A
(different blot with the same samples from above)

Figure 4B

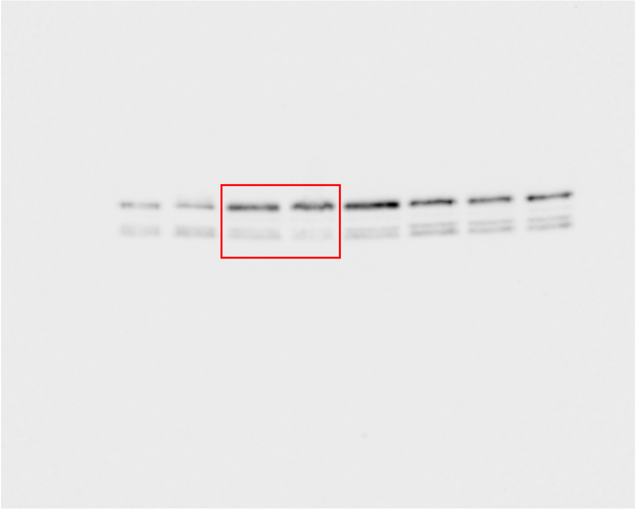

IB: WDR1
WCE from cells expressing STK16 WT/E202A

Figure 4B

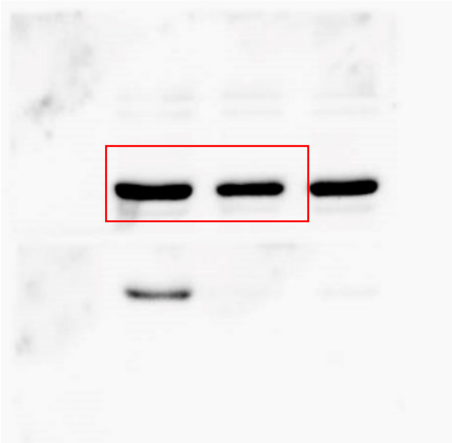

IB: tubulin
WCE from cells expressing STK16 WT/E202A

Figure 4D

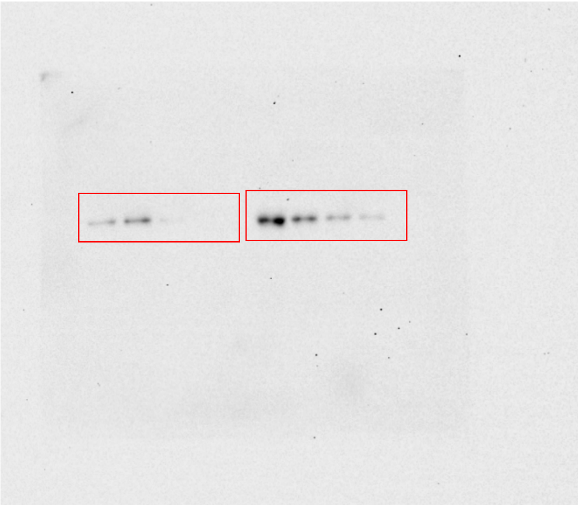

IB: anti-WDR1
Elutions 1 - 4 from control on left
(top panel in figure)
Elutions 1 - 4 from OA-treated cells on right
(bottom panel in figure)

Figure 4E

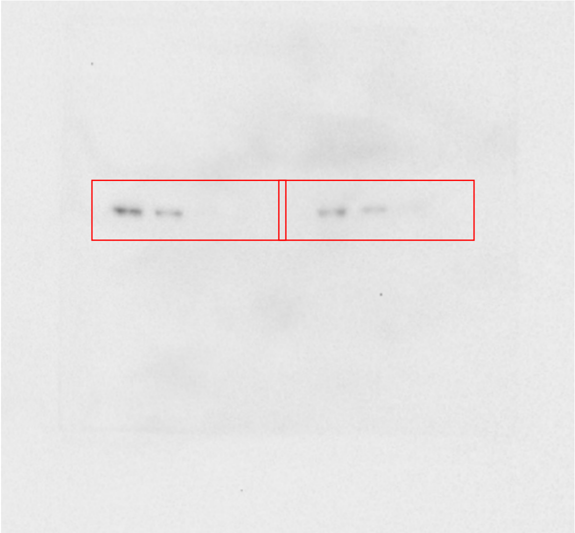

IB: anti-WDR1
Elutions 1 - 4 from WT expressing cells on left
(top panel in figure)
Elutions 1 - 4 from E202A expressing cells on right
(bottom panel in figure)

Figure 5C

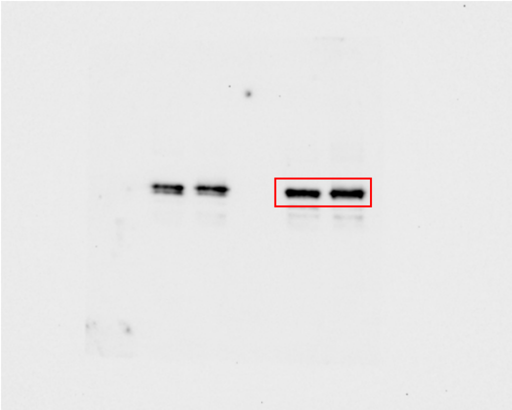

IB: anti-WDR1
WCE from control cells/OA-treated cells

Figure 5C

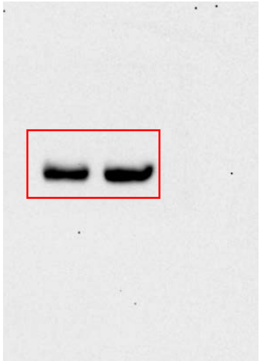

IB: anti-tubulin
WCE from control cells/OA-treated cells

Figure 6D top panels (short)

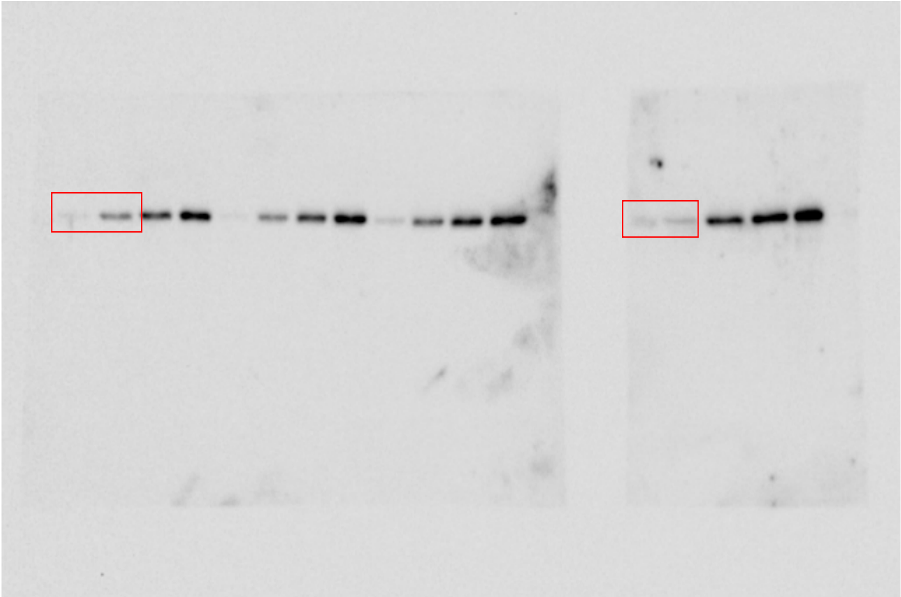

IB: anti-albumin
Secretion assay
0 and 15 min time points
From control on left (bottom left panel in figure)
From cells treated with “short” lat B on right (bottom left panel in figure)

Figure 6D bottom panels (long)

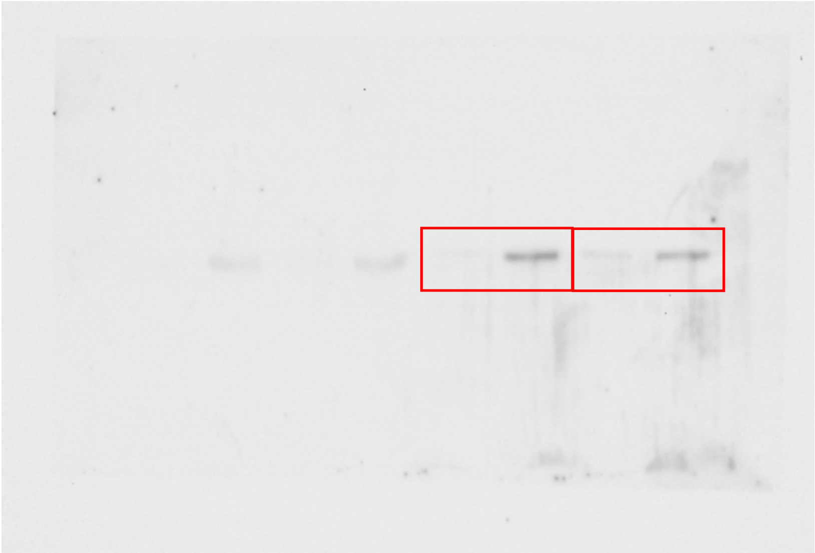

IB: anti-albumin
Secretion assay
0 and 15 min time points
From control on left bottom right panel in figure)
From cells treated with “long” lat B on right (bottom right panel in figure)

Figure 6D top panels (short)

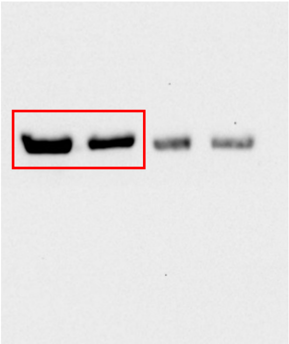

IB: anti-tubulin
Secretion assay
Whole cell lysates from control on left or “short” lat B on right (top left panel in figure)

Figure 6D top panels (long)

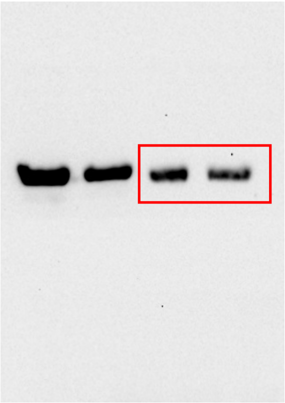

IB: anti-tubulin
Secretion assay
Whole cell lysates from control on left or “long” lat B on right (top right panel in figure)

Figure 7B

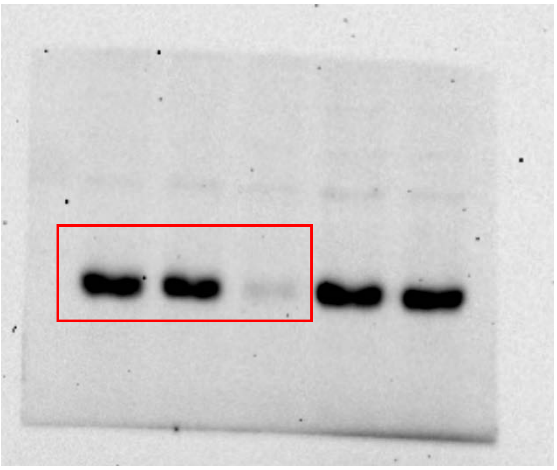

Top Panel:
IB: anti-WDR1
Cells treated with con, scrambled or WDR1 siRNA

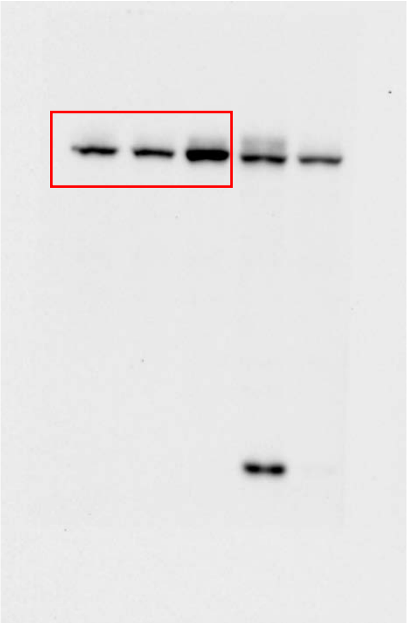

Middle panel:
IB: anti-V5 (to detect sDPPIV)
Cells treated with con, scrambled or WDR1 siRNA
(same samples as above)

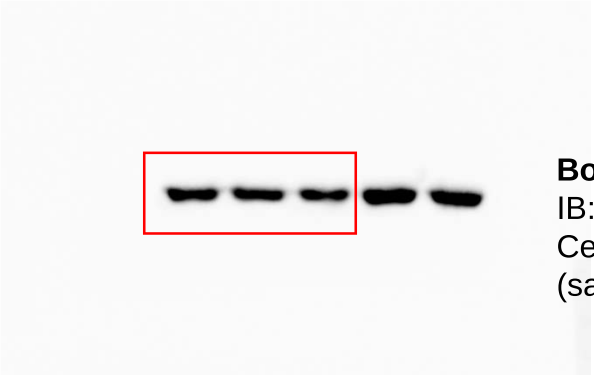

Bottom panel:
IB: anti-a-tubulin
Cells treated with con, scrambled or WDR1 siRNA
(same samples as above)

Figure 7B

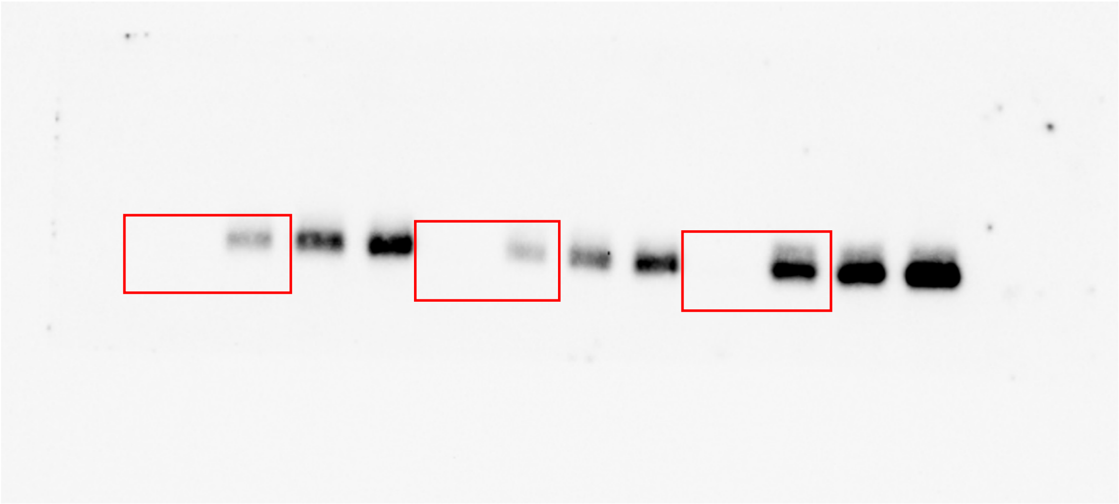

IB: anti-V5 (to detect sDPPIV)
Secretion Assay
0 and 60 min time points
From control (untransfected) cells on left (top panel in figure)
Treated with scrambled siRNA in middle (middle panel in figure)
Treated with WDR1 siRNA on right (bottom panel in figure)
